# Safety and efficacy of the PrePex device in HIV-positive men: A single-arm study in Zimbabwe

**DOI:** 10.1371/journal.pone.0189146

**Published:** 2017-12-08

**Authors:** Mufuta Tshimanga, Batsirai Makunike-Chikwinya, Tonderayi Mangwiro, Patricia Tapiwa Gundidza, Pesanai Chatikobo, Vernon Murenje, Amy Herman-Roloff, Peter H. Kilmarx, Marrianne Holec, Gerald Gwinji, Owen Mugurungi, Munyaradzi Murwira, Sinokuthemba Xaba, Scott Barnhart, Caryl Feldacker

**Affiliations:** 1 Zimbabwe Community Health Intervention Project (ZiCHIRe), Harare, Zimbabwe; 2 International Training and Education Center for Health (I-TECH), Harare, Zimbabwe; 3 Department of Health Sciences, University of Zimbabwe, Harare, Zimbabwe; 4 U.S. Centers for Disease Control and Prevention, Harare, Zimbabwe; 5 International Training and Education Center for Health (I-TECH), Seattle, Washington, United States of America; 6 Ministry of Health and Child Care, Harare, Zimbabwe; 7 Zimbabwe National Family Planning Council (ZNFPC), Harare, Zimbabwe; 8 Department of Medicine, University of Washington, Seattle, Washington, United States of America; 9 Department of Global Health, University of Washington, Seattle, Washington, United States of America; University of Ottawa, CANADA

## Abstract

Male circumcision (MC) for sexually active, HIV-negative men reduces HIV transmission and averts HIV infections. Excluding HIV-positive men from MC decreases access to additional health and hygiene benefits. In settings where HIV-testing is, or is perceived to be, required for MC, testing may reduce MC uptake. Reducing promotion of HIV testing within MC settings and promoting device-based MC may speed MC scale-up. To assess safety and efficacy of PrePex MC device among HIV-positive men, we conducted a one-arm, open-label, prospective study in otherwise healthy HIV-positive men in Zimbabwe.

**Methods:**

We aimed to determine if the adverse event (AE) rate was non-inferior to an AE rate of 2%, a rate considered the global standard of MC safety. Study procedures, AE definitions, and study staff were unchanged from previous PrePex Zimbabwe trials. After PrePex placement and removal, weekly visits assessed wound healing. Men returned on Day 90. Safety was defined as occurrence of moderate and serious clinical AEs. Efficacy was defined as ability to reach the endpoint of complete circumcision.

**Results:**

Among 400 healthy, HIV-positive, consenting adults, median age was 40 years (IQR: 34, 46); 79.5% in WHO stage 2; median CD4 was 336.5c/μl (IQR: 232, 459); 337 (85%) on anti-retroviral therapy. Among 385 (96%) observed completely healed, median days to complete healing was 42 (IQR: 35–49). There was no association between time to healing and CD4 (p = 0.66). Four study-related severe AEs and no moderate AEs were reported: severe/moderate AE rate of 1.0% (95% CI: 0.27% to 2.5). This was non-inferior to 2% AEs (p = 0.0003). All AEs were device displacements resulting in surgical MC and, subsequently, complete healing.

**Conclusion:**

Male circumcision among healthy, HIV-positive men using PrePex is safe and effective. Reducing the barrier of HIV testing while improving counseling for safer sex practices among all MC clients could increase MC uptake and avert more HIV infections.

## Background

Male circumcision (MC) reduces the risk of female-to-male HIV-1 transmission by 60% [1–3]; millions of new HIV infections, and subsequent deaths, could be averted if MC coverage is reached [4]. Surgical MC has proven safe in both controlled and field settings [1–3, 5–11]. From 2008–2015, nearly 14.5 million voluntary medical male circumcision (VMMC) procedures were performed across 14 African countries [12], shy of the 20 target million needed [13] to avert 3.4 million HIV infections and save $16.5 billion in HIV-related care through 2025 [14].

Non-surgical MC devices, such as PrePex, have the potential to accelerate VMMC program scale up. In pilot studies and controlled trials among HIV-negative men in sub-Saharan Africa, PrePex has proven safe and acceptable with moderate and severe adverse event (AE) rates (most commonly from device displacement, swelling, pain) from 0–5.9% [6, 9, 15–20]. Globally, few reported AEs from any method of MC, including PrePex, resulted in permanent impairment or death. PrePex is considered faster, simpler to implement, and, with some exception [21], may be more cost effective when compared to surgical MC [22–24]. PrePex may be safely applied by less highly trained cadres of nurses and clinicians [19, 25, 26]. In some contexts, devices appear more acceptable than surgical MC as they do not cause prolonged pain [6] nor interrupt activities of daily living for most clients [18]. For these reasons, PrePex offers an important tool to accelerate VMMC programs to meet targets especially in men over age 18 years.

VMMC is almost-exclusively promoted for sexually active, HIV-negative men as part of comprehensive HIV prevention [13]. To reach HIV-negative men, VMMC is commonly accompanied by HIV testing. HIV-related stigma, however, may reduce acceptance of HIV testing [27] and, therefore, VMMC uptake in sub-Saharan Africa [28–30]. Recent modeling shows that inclusion of all men regardless of HIV status may significantly increase the effectiveness of VMMC scale up by reaching more at-risk males, potentially improving the pace of HIV infection prevention in countries most in need [31]. Moreover, there are additional benefits to adult male circumcision including improved hygiene [32], reduction in penile cancers [33, 34], and decreases in some sexually transmitted diseases [35, 36], advantages of which some may apply to HIV-positive men [37]. Although HIV-positive men have largely been ignored in both surgical and PrePex MC scale-up efforts [38], access to VMMC is desirable for all men regardless of HIV status.

Surgical VMMC appears safe in otherwise healthy, HIV-positive men [8, 39–41]. Yet, research on the safety of use of MC devices such as PrePex in HIV-positive men is very limited. This lack of evidence is most acute where rapid expansion of male circumcision programs is most urgent, including Zimbabwe. Although HIV testing is not a precondition to being circumcised in Zimbabwe, it is strongly recommended as part of the minimum package of essential HIV prevention services [42, 43]. Virtually all men in Zimbabwe are HIV tested prior to circumcision [44, 45]. Although HIV testing is an important component of combination HIV prevention, qualitative research in Zimbabwe found that the presumption of HIV testing prior to VMMC presents a formidable barrier to VMMC among some men due to fear of, or reluctance to, HIV test. [46, 47]. Quantitative studies, also from Zimbabwe, further support the strength of this persistence barrier, finding that between 36% [48] and 50% [49] of men noted that HIV testing before the procedure was a reason to avoid VMMC. If, however, surgical and device-based VMMC is safe for HIV-positive men, then the need for HIV testing before VMMC is reduced. Revised messaging to promote the availability of, but not requirement for, HIV testing within VMMC settings could diminish the perceived mandate for HIV testing, thereby increasing VMMC uptake among all men. As Zimbabwe’s progress towards national VMMC goals is slower than expected [50], aiding demand for VMMC could speed efforts to avert new HIV infections. Increased risk reduction counseling on the importance of abstinence or condom use during the healing period for all men regardless of HIV status would be beneficial in conjunction with lessened emphasis on HIV testing before VMMC.

In order to assess the safety and efficacy of the PrePex device in HIV-positive men, we conducted a one-arm, open-label, prospective study in otherwise healthy HIV-positive adult men in Zimbabwe. If PrePex proves safe for use among HIV-positive men, PrePex could be promoted to all men regardless of HIV status, including those with unknown status. Therefore, results from this study may inform policy and clinical practice pertaining to HIV testing among men seeking circumcision (S1 Table).

## Methods

### Study design and sample size

This study aimed to determine if the AE rate among the study population, composed of HIV-positive men who receive PrePex MC, was non-inferior to that of the historical proportion of men who experience an AE with surgical MC of 2% [1–3]. The historical AE rate of 2% was selected as it is generally consistent with the AE rates of previous MC randomized control trials [1–3] and because an AE rate of ≤2% is regarded both as an acceptable global standard of VMMC safety and the adopted standard of care for Zimbabwe [51–53]. The non-inferiority margin, based on statistical and clinical considerations, is the maximum difference between the rate of AEs among HIV-positive men using PrePex and historical controls where we would conclude that PrePex among HIV-positive men is not inferior to routine care. That margin was set at 2%, determining that a 4% AE rate would be non-inferior. Including a possible 20% lost to follow up, 400 participants per arm provides 80% power at an alpha of 2.5% to detect if AEs exceeded the non-inferiority margin of 2%. The study protocol is available in S1 Text. The trial is registered at ClinicalTrials.gov (STUDY00000126).

### Study team

Procedures were conducted by two surgeons (National PrePex Master trainers) and three nurses who had undergone PrePex training in 2011, were involved in previous PrePex studies in 2011 and 2012, and had additional training for the current study. The initial 50 PrePex circumcisions were conducted by the surgeons; the remaining 350 circumcisions were conducted by the nurse providers.

### Study site and recruitment

The study was implemented in Chitungwiza, a large urban area outside of Harare by the same team comprised of the same cadres of staff that carried out previous PrePex studies in Zimbabwe, mentioned above. For staff, brief training included updates on the minimum package of VMMC services, the potential for increased risk of HIV transmission to providers and partners, and the need to maintain expected standards of strict infection control measures. Participant recruitment from HIV treatment clinics sites was conducted from October 2015 to May 2016; follow-up lasted 90 days. Nurses at these clinics discussed the study about MC for HIV-positive men during routine education sessions for all HIV-positive men; any interested men traveled independently or with study-provided transport to Seke South Clinic for further assessment. This process also reduced possible stigma of participants or the association of the VMMC clinic with only HIV-positive men.

At Seke South, any males aged 18 and above interested in circumcision who accepted to be HIV tested, and who tested HIV-positive, could participate in the study. This included men who initially heard about the study at HIV treatment clinics or who independently came for VMMC at Seke South. Further inclusion criteria included: confirmed HIV-positive status; WHO HIV clinical stage 1 or 2; agreed to be circumcised using PrePex; agreed to return to the health care facility for study follow-up visits; and agreement to anonymous photographs. Exclusion criteria included: known bleeding or coagulation abnormality; uncontrolled diabetes; active genital infection; or anatomic abnormality. Potential participants who did not meet the inclusion criteria were offered surgical circumcision.

### Study procedures

All participants who consented to study participation received the minimum package of VMMC services that included information about the risks and benefits of the MC procedure, HIV testing, and management of sexually transmitted infections (if found during VMMC eligibility examination) before the procedure. Participants were also reminded about the added risk of HIV infection to their partners if they resumed sex before complete healing and received counselling about the need to adopt and maintain safer sexual practices, complemented by condom promotion during all post-procedure follow-up visits. CD4 data from participants were collected using a small finger prick to obtain blood sample and analyzed using PIMA Point of care CD4 machine.

After placement at Day 0 and removal at Day 7, additional weekly visits for clinical review were scheduled for all participants until the wound was assessed as healed. Participants were also asked weekly about resumption of sexual activity until they were assessed as healed. If a participant failed to attend the scheduled weekly visit, up to three contact attempts were made by phone and SMS. For a missed Day 7 device removal visit, the study nurse also attempted at least two home visits. In addition, participants were instructed to return to the clinic for an unscheduled visit if they experienced any AEs or complications with the device. All men were asked to return for a Day 90 visit for a final physical exam. All participants were reimbursed US $6 for travel for each study visit.

## Data management

### Definitions

**Safety** was defined as the occurrence of any moderate and serious clinical AEs as defined in previous clinical trials in Zimbabwe [54] including the following AE types, among others: site bleeding, penis-diffused hematoma, penis-diffused edema, and incision site infection (S2 Text). If two AEs occurred in the same individual, the more severe AE was considered in the safety analysis. Expected, mild side effects such as minor localized edema, oozing, and clear exudates are not included in the primary safety endpoint. **Efficacy** was defined as fully healed without surgical intervention at the end of the study. **Time to complete wound healing** was defined as the number of days from the date of device placement (not from the date of intended or actual device removal) to the first date when complete wound epithelialization was observed[55].

### Analysis

Data were analyzed using STATA 12.0 [56]. Binomial probability tests evaluated equivalence of proportions; Clopper-Pearson exact confidence intervals were calculated.

### Ethical conduct of the study

Institutional review boards approved the study at the Medical Research Council of Zimbabwe (MRCZ); the University of Washington; and the U.S. Centers for Disease Control and Prevention (CDC). Participants voluntarily provided written informed consent. A Data Safety Monitoring Board of Zimbabwean medical experts met four times over the course of the study to provide oversight in protocol adherence, patient safety, and data quality and in all phases of the trial. This manuscript was reviewed for clearance by CDC.

## Results

### Recruitment, screening, eligibility and demographic characteristics

**Fig 1:**
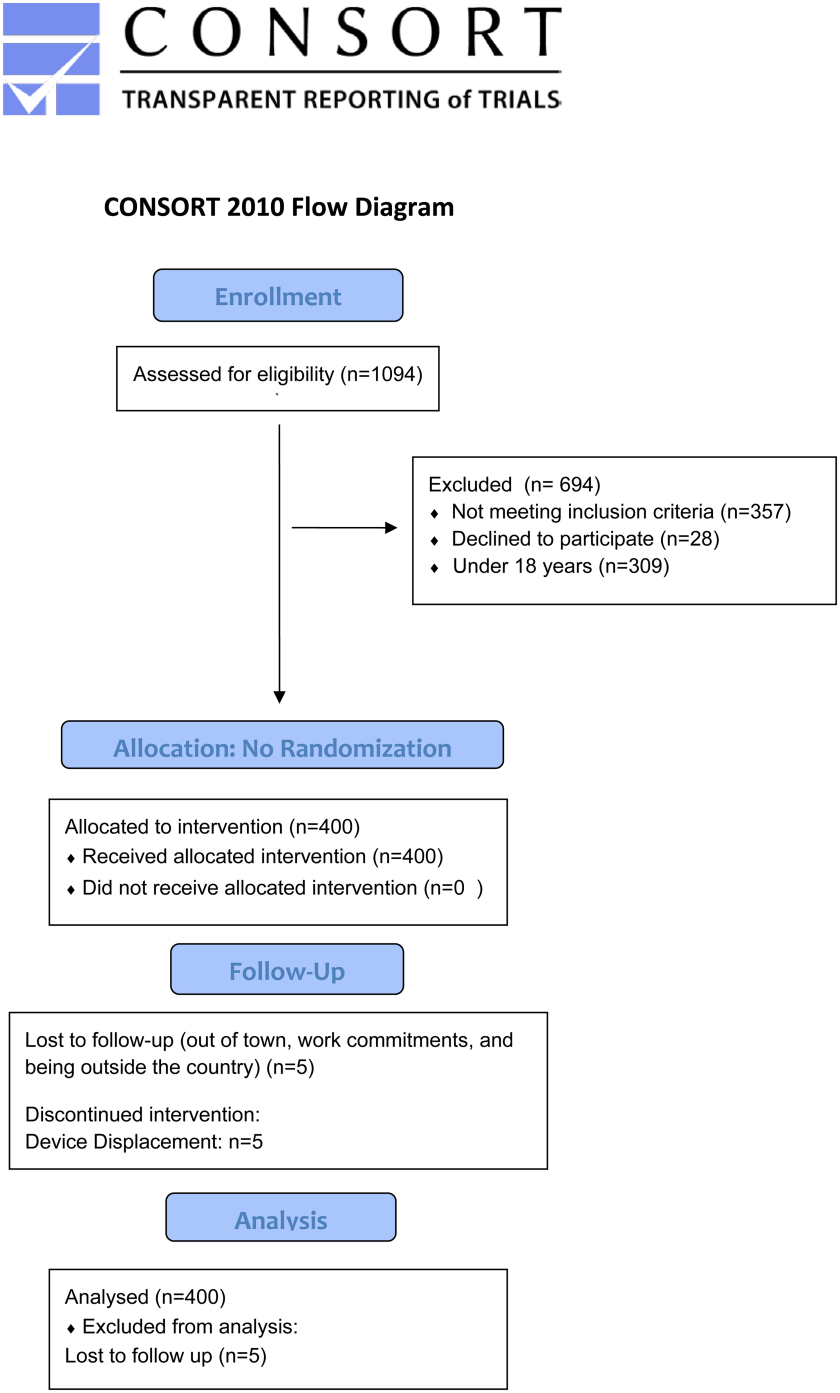
CONSORT flow diagram.

A total of 1094 clients were recruited (Fig 1); 309 were under age 18. Of adult men, 28 declined (2.6%), showing that most HIV-positive men interested in VMMC as part of the study found PrePex to be acceptable. Of 757 adults screened, 400 were eligible. Table 1 also details ineligibility among those who met overall inclusion criteria.

**Table 1:** Study flow and reasons for non-eligibility of participants.

Study component	N			
Recruited	1094			
Excluded <18 years	309			
Declined participation	28			
Screened but excluded	357	Reason:	N	%
		HIV negative	292	81.8%
		Genital warts	10	2.8%
		Genital ulcer	6	1.7%
		Adhesions	6	1.7%
		Phimosis	5	1.4%
		Hypertensive	5	1.4%
		Keloids	4	1.1%
		Unable to come for reviews	4	1.1%
		Opted for surgical MC	4	1.1%
		Genital sores	3	0.8%
		Uncontrolled diabetes	3	0.8%
		WHO Stage 3 or 4	3	0.8%
		Already circumcised	3	0.8%
		Hydrocele	2	0.6%
		Enlarged prostate	2	0.6%
		Refused HIV testing	2	0.6%
		Urethral discharge syndrome	1	0.3%
		Epistaxis	1	0.3%
		Hypospadias	1	0.3%
**Enrolled study participants**	**400**			

Of the 400 eligible participants, the median age of participants enrolled in the study was 40 years (rage: 18–70) (Table 2). The majority of participants were in WHO stage 2 (79.5%). Median CD4 was 336.5c/μl, with a range of 8 to 1050 c/μl. Almost 85% of men (337/400) were on anti-retroviral therapy (ARVs) at the time of MC. Most (75.2%) participants came from Chitungwiza.

**Table 2:** Participant demographics (N = 400).

Characteristic (Mean; *IQR)	Frequency	Proportion (%)
Age (40.3; 33.5–46.0)		
<25	18	4.5
25–34	87	21.8
35–44	173	43.3
45+	122	30.5
CD4 (352.1; 232–459)		
0–199 c/μl	76	19.0
200–499 c/μl	249	62.3
≥500 c/μl	75	18.7
WHO clinical stage		
Stage 1	82	20.5
Stage 2	318	79.5
ARV regimen		
1^st^ line	322	80.5
2^nd^ line	15	3.7
None	63	15.8
Marital Status		
Married	306	76.5
Single	44	11.0
Separated / Divorced	29	7.3
Widowed	21	5.3
Type of employment		
Informal	210	52.5
Formal	118	29.5
Unemployed	65	16.3
Student	7	1.8
Residence		
Chitungwiza	301	75.2
Other	99	24.8

*IQR = Interquartile range

### Device removal

The vast majority (394 (98.5%)) of devices were successfully removed on-site on Day 7 following placement. For the six other participants, one removed their device within hours of placement and no further intervention was required; one participant reported removal on day 7 at another site; 4 others were considered AEs and described below.

### Adherence to study visits

Participants were expected to attend all scheduled visits according to the study schedule until complete healing was assessed. As noted previously, 98.5% of men attended the Day 7 removal visit; 96.5% attended the 14-day visit. Thereafter, weekly visit adherence through Day 42, the final day of assessment for routine PrePex VMMC, was 93% or greater at each time point. Visit adherence among those not assessed as healed dropped to 88.3, 77.4, and 47.6 for Days 49, 56, and 63, respectively. All participants were requested to return for the Day 90 study closeout visit: final study visit attendance was 94.2%. All men who did not report to the final visit were contacted by phone and reported being healed. Reasons for missed reviews included being out of town, work commitments, and being outside the country.

### Time to complete healing

PrePex proved efficacious: 385 (96%) were observed as being fully healed without surgical intervention. Time to complete healing for those 385 participants who attended review visits at the scheduled time and presented by study visit date (Table 3). Assessment of complete healing was established if the scar was soft and pliable upon pinching and a stretching test showed no breach. Ten participants missed their review visits before complete healing could be observed but reported full healing via study follow-up call. As described above, four men were surgically circumcised after device displacement and one man was not circumcised. Median days to complete healing was 42 days (IQR: 35–49; range 28–70); mean time to healing was 42.3 days. Eighty-eight percent of men were fully healed by Day 49. Only four men (1.0%) reported having sex before they were observed as fully healed; three of the four were on ARVs. There was no significant association between time to complete healing and CD4 (p = 0.66) (Table 3) nor time to complete healing and ARV status (p = 0.17).

**Table 3:** Percent of men healed at each visit, by CD4 (N = 385).

Visit date*	CD4			Row Total	Row %	Cumulative %
	0–199	200–499	≥500			
	N (%)	N (%)	N (%)			
≤Day 28	7 (10.0)	12 (5.0)	6 (8.2)	25	6.5	6.5
Days 29–35	20 (28.6)	65 (26.9)	17 (23.3)	102	26.5	33.0
Days 36–42	19 (27.1)	74 (30.6)	26 (35.6)	119	30.9	63.9
Days 43–49	18 (25.7)	59 (24.4)	14 (19.2)	91	23.6	87.5
Days 50–56	3 (4.3)	20 (8.3)	9 (12.3)	32	8.3	95.8
Days 57–63	3 (4.3)	11 (4.5)	1 (1.4)	15	3.9	99.7
>Day 63	0	1 (0.4)	0	1	0.3	100.0
Total	70 (18.2%)	242 (62.9%)	73 (19.0)	385		

*Days post placement

### Adverse events

There were four cases of expected side effects: all four were cases of localized edema managed by penile elevation. No moderate AEs were reported.

A total of four study-related severe AEs were reported resulting in a severe AE rate of 1.0% (95% CI: 0.27% to 2.5%). The study’s AE rate of 1% is less than or equal to the 4% upper bound for safety as determined for non-inferiority as defined in this study (p = 0.0003).

AE details are provided in S3 Text. Four device displacements, when the PrePex device was intentionally (1 case) or accidentally (3 cases) dislodged during the 7-day placement period, were reported: one on day 1; two on day 3; and one on day 5. All device displacements were resolved with surgical circumcision. These are considered severe because a surgical MC procedure is required as part of clinical management. Due to their HIV-positive status, and at the discretion of the treating clinician, three of these men were given oral antibiotics after surgical MC as a precaution against infection. All four men healed completely. Additionally, two non-study related severe AEs were reported and not included in the study-related AE rate. One urethral stricture detected on day 37 was determined to be a pre-existing condition the client first noted, but did not initially report, September 2015, and resolved after bladder catheterization. One study participant death was reported due to car accident on day 35, unrelated to the study. There were no cases of partial or complete glans removal, penile loss, or study-related deaths.

### AEs among Zimbabwe historical controls

The study qualitatively compare the AE results to three previous PrePex device trials in Zimbabwe led by the same study team (Table 4). In Phase 1, the initial safety trial in Zimbabwe conducted in October-November, 2011, 53 HIV-negative adults underwent PrePex; 3 mild AEs were found [57]. Phase 2, November, 2011- January, 2012, compared PrePex and forceps guided VMMC among HIV-negative adults[22] finding no AEs among 80 forceps guided clients and 2 mild AEs (1.3%) among 158 PrePex clients (95% CI: 0.003–4.53%). In Phase 3, 603 adult men had PrePex placed by registered general nurses; 4 men (0.7%) had moderate AEs (1 urethral cut at removal; 1 difficult removal requiring sutures; 1 wound gaping, and 1 device displacement) that resolved within the expected healing period[58]. Lastly, recent VMMC program data on PrePex and surgical clients in Zimbabwe from October 2014 to September 2015, found 0.3% of surgical and 1.2% of PrePex clients experienced a moderate or severe AE [59], with PrePex AEs commonly due to device displacements (70%), infection (20%), and pain (12%).

**Table 4:** Summary of AEs reported in previous PrePex studies in Zimbabwe.

	Number of MCs		Moderate/Severe AEs	
Comparative data	Surgical	PrePex	Surgical	PrePex
Phase 1 trial	-	53	n/a	0
Phase II trial	80	158	0	0
Phase III trial	-	603	n/a	4 (0.7%)
VMMC program data*	41416	3452	116 (0.3%)	40 (1.2%)

* [59]

## Discussion

This study demonstrated that PrePex is safe for healthy, HIV-positive men in WHO stage 1 or 2. With a moderate/severe AE rate of 1.0%, the risk of AEs in this study is not different from the AE rate from previous PrePex trials and routine program delivery among HIV-negative men within Zimbabwe [22, 57–60] nor does it differ from rates found in other previous PrePex [17] or devices studies [61] among HIV-negative men in the region. Additionally, there were no moderate AEs reported and the severe AEs noted in the study were device displacements which resulted in correction with surgical MC, the MC method they would have in the absence of PrePex. Although the study’s AE rate is higher than the all-method, national VMMC program AE rate of 0.35% [53], it is non inferior in terms of safety as compared to the accepted AE risk of ≤2%. With adherence to follow-up visit at Day 14 of almost 97%, it is unlikely that additional severe or moderate AEs were missed since most AEs from surgical MC are also expected before Day 14 [5, 62], and over 95% of all AEs within routine VMMC service delivery are reported by Day 14 [63]. Previously, only known HIV-negative men were candidates for PrePex. The findings of this study support extension of PrePex for healthy HIV-infected men as well as healthy men who refuse HIV testing and, therefore, have unknown HIV status. Counseling for, and access to, HIV testing should remain a core component of combination HIV prevention [64].

Prior to this study, there was no published data on time to healing among HIV-positive men using PrePex. This study demonstrated that PrePex is efficacious for HIV-positive men. Of the 400 men, 385 were observed as fully healed using the PrePex method (96%); 10 more men reported complete healing by phone, bringing efficacy to 99% (395/400). The mean time for healing among the 385 men was 42 days after placement, similar to healing time from PrePex prequalification (44.1 days) [65] and within the healing range of PrePex studies among HIV-negative men with means of 32.3 [17] and 48 days post placement [9]. As compared to previous studies conducted by the same study team among HIV-negative men, median time to complete healing was the same at 42 days post placement [57, 58, 60]. However, the proportion of men fully healed by Day 42 in this study of 64% is lower than the 79.2% [57], 87% [22], and 80.2% [58] found among HIV-negative men, suggesting a slower pace of healing for HIV-positive men. During this extended healing period, these men may be shedding virus and, potentially, increasing HIV transmissions. This possibility must be effectively addressed through improved counseling on abstinence until fully healed especially for HIV-infected or HIV-unknown men.

Although healing may be slower among HIV-positive men who have PrePex VMMC, CD4 count was not related to time to complete wound healing. In accordance with Zimbabwe policy, the current VMMC program Client Intake Form ascertains HIV status and includes both CD4 and WHO stage as part of the medical screening and physical examination. Only WHO staging is required before VMMC: those in Stages I and II may be circumcised while men in Stages 3 and 4 require care and treatment before VMMC. CD4 count is not mandatory but can be used if available. These results suggest WHO clinical staging may be a better determinant of a client’s eligibility for safe circumcision over CD4 and would address the lack of CD4 services in low resource settings where both HIV burden and demand for HIV prevention services, like VMMC, is high. If the results of this study are leveraged to reduce the barrier of HIV testing and, therefore, increase VMMC uptake among HIV-positive men or men with unknown HIV status, it is possible that HIV-positive, asymptomatic, WHO Stage 3 men could get circumcised. As all men receive a thorough exam and medical history as part of VMMC eligibility, and all VMMC programs assess AEs as part of routine follow-up, these standard practices appear sufficient to maintain client safety.

These results on time to healing among HIV-positive men after PrePex MC and lack of relationship between CD4 and healing time echoes the results of surgical MC studies. A study in Kenya using the forceps-guided method among adult men found no statistically significant difference in mean time to complete healing: 33 days for HIV-positive vs. 31 days for HIV-negative [41], but, unlike this study, did find a difference by CD4 status with mean time to complete healing of 37.1 days among men with CD4<350 as compared 31.1 days among those with a CD4 count greater than 350 (p = 0.04). In Uganda, a study using the surgical sleeve method found that more than 90% of wounds healed by 6 weeks post-MC; however, HIV-negative men healed slightly faster: complete wound healing was certified in 73% of HIV-positive men and 83.2% of HIV-negative men (p<0.001) at 30 days post-MC [39]. Another study in Uganda using surgical dorsal slit method among men ages 12 and older found no significant difference in time to healing by HIV or CD4 status [66]. In summary, wound healing for those who are HIV-positive, including those with CD4 <350, may be slightly longer than for those who are HIV-negative, suggesting the need for increased HIV prevention counseling for men on the important of abstinence or condom use until complete healing is confirmed.

Although there is some hesitation to circumcise HIV-positive men due to a perceived higher risk of HIV transmission to partners during the healing process, in this study, most were on ARVs at the time of MC and only four men, three of whom were on ARVs, reported having sex the week before they were fully healed. It appears that most men abstained from sex until complete healing as instructed, reducing risk to partners. Moreover, the Zimbabwe Population-Based HIV Impact Assessment (Zimphia) 2015–2016 found that of men who knew their HIV status, 86.0% reported taking ARVs, and 84.1% of men on ARVs reported being virally suppressed [67], potentially reducing the risk of transmission during the healing process. Men may underreport sexual activity; however the overall risks to female partners, especially during the healing process, are difficult to quantify and remain unclear [68–70]. Although more research is needed, the risk of potential HIV transmission to female partners may not be a strong enough argument against circumcising HIV-positive men or those with unknown status within the VMMC program. What is clear is that men who undergo PrePex MC, especially those who are HIV-positive, must be well counselled to abstain from sex or to use a condom for both HIV transmission prevention and wound protection for at least seven weeks post device placement or until fully healed [65].

It is critical to note that the benefits of VMMC extend beyond HIV, benefits that may extend to HIV-positive men. VMMC brings hygienic benefits, an opinion shared by both men and women [32] and protects males from cancers of the penis, urinary tract infections and ulcerative sexually transmitted diseases [71–73]. VMMC also improves penile hygiene and reduces the risk of balanitis, which also affect HIV-positive men. VMMC reduces the prevalence and incidence of human papilloma virus infections in female partners [37, 69, 74, 75], a benefit that may also reach partners of HIV-positive men [37], decreasing the risk of cervical cancer among these women.

This study has several limitations. First, this study compared the AE rate to historical controls in the same facility using the same providers and to the global acceptable standard of AEs of ≤2%; there is no within-study comparison group for this study. However, because we looked at both the previous studies’ AE rates using the same study team and also looked at AE rates among HIV-negative men in a large, routine VMMC program in Zimbabwe, we believe the results of this one-arm study overcome this weakness. Moreover, the AE definitions and severity categorizations used in this study are less conservative than the revised AE guidelines used in the U.S. President′s Emergency Plan for AIDS Relief (PEPFAR) programs as of 2013, potentially leading to reductions in diagnosis or reporting of AE severity in this study. However, using the revised AE guidelines to review the data[76] and applying the most conservative definitions, three additional moderate AEs (infections) could be considered as three of the four device-related AEs cases were given antibiotics as potential infection could not be confidently ruled out, leading to an AE rate of 1.8% (7/400). This would not alter the study conclusions. Men in this study were recruited from HIV treatment clinics, increasing the likelihood that they were on ARVs. Lastly, this study did not collect variables related to HIV transmission (viral load, viral shedding, condom use) nor enroll female partners for longitudinal follow-up. Despite these limitations, we believe results support the safe, effective use of PrePex among HIV-infected men.

## Conclusion

Male circumcision among healthy HIV-positive men using the PrePex device is safe. The risk of adverse events is not different than the ≤2% rate used as a standard of quality care for MC practice. The only AEs found in this study were device displacements. Also, this study included men with low CD4; yet, CD4 did not appear a factor in the occurrence of study-related AEs. Therefore, it appears that WHO clinical staging is sufficient for VMMC inclusion criteria for HIV-positive men, reducing the need for CD4 testing before VMMC. We strongly support both HIV testing and VMMC as critical components of comprehensive HIV prevention. However, as PrePex appears safe for all men regardless of HIV status, it is important to weigh promotion of HIV testing against the benefits of VMMC for men who might otherwise shy away from MC services fearing HIV test results. Therefore, we recommend that HIV testing be advertised to potential VMMC clients as, “recommended, not mandatory”. In concert with continued promotion of safer sex practices in line with VMMC guidelines and increased risk reduction counseling for abstinence or condom use until completely healed for all men, we expect that service provision could be made more efficient and VMMC uptake increase as a result of this suggested policy change, averting additional HIV infections in the region.

## Supporting information

S1 Table Trend checklist. (DOCX)

S1 Text Study protocol. (DOCX)

S2 Text Zimbabwe AE definitions. (DOCX)

S3 Text Details on study-related AEs. (DOCX)
