# Supplementary material for: Safety and efficacy of the PrePex device in HIV-positive men: A single-arm study in Zimbabwe
Source: PLoS One. 2017 Dec 8;12(12):e0189146. doi: 10.1371/journal.pone.0189146 (PMC5722373; doi:10.1371/journal.pone.0189146)
Supplement: S1 Text — (DOCX) [file pone.0189146.s002.docx]

Rev - 19th June 2012

MRCZ/A/1628

**PrePex MC**

**Classification of Adverse Events and Device Hazards**

Adverse

Event

Description Severity Code

A. During Placement

Pain Pain score of 8 or more not requiring anesthesia

Requires anesthesia

Not controlled by additional anesthesia

Mild

Moderate

Severe

APP1

APP2

APP3

Difficulty in applying the device

Could not apply device; determined as contra indicated;

no harm to tissue or subject

Had to push unusually hard, but no harm to tissue or subject and no change to procedure

The device cut through the foreskin, with or without minor bleeding, No change in procedure

The device cut through the foreskin, with or without minor bleeding, and requiring a change to surgical method

The device cut through the foreskin, causing significant bleeding and requiring a change to surgical method

No AE No AE Mild Moderate

Severe

--

-- ADD1

ADD2

ADD3

B. While Wearing the Device

Pain / Discomfort Not requiring intervention beyond painkiller or anesthetic cream

Requiring early device removal by PrePex Operator or by client

Requiring early device removal and anesthesia

Not controlled by device removal or additional anesthesia

Not AE Mild

Moderate

Severe

-- BWP1

BWP2

BWP3

Device displacement / spontaneous detachment

Device displacement with no clinical consequences Complete spontaneous detachment, or patient removed device himself with no adverse clinical consequences

Device displacement requiring re-placement

Device displacement requiring surgical intervention

Displacement or detachment and penile damage present

Not AE

Mild

Moderate

Severe

--

BDD1

BDD2

BDD3

Early device removal (i.e. 4 days or less with the device)

Device removed due to pain, swelling or bleeding

Device removed due to pain, swelling or bleeding requiring surgical intervention

Device removed and penile damage present

Mild

Moderate

Severe

BER1

BER2

BER3

Edema More edema than usual but not causing any discomfort to the patient

Moderate edema causing the patient discomfort, though managed with conservative measures

Severe edema, causing the patient discomfort, uncontrolled with conservative measures

Mild

Moderate

Severe

BED1

BED2

BED3

Hematoma Mild contained hematoma, not requiring any treatment

Hematoma requiring surgical drainage/exploration but no evidence of active bleeding

Rapidly expanding hematoma suggesting active bleeding requiring surgical exploration or referral

Mild

Moderate

Severe

BHM1

BHM2

BHM3

C. During Device Removal

Pain Pain score of 6 or less lasting for less than 2 minutes Pain score of 8 or more lasting for over 2 minutes Requires anesthesia

Not controlled by additional anesthesia

Not AE Mild Moderate

Severe

-- CPR1

CPR2

CPR3

Excessive bleeding More bleeding than usual, but easily controlled

Bleeding that requires suture to control

Blood transfusion or transfer to another facility for management required

Mild Moderate Severe

CBL1

CBL2

CBL3

Edema More edema than usual but not causing any discomfort to the patient

Moderate edema causing the patient discomfort, though managed with conservative measures

Severe edema, causing the patient discomfort, uncontrolled with conservative measures

Mild

Moderate

Severe

CED1

CED2

CED3

Hematoma Mild contained hematoma, not requiring any treatment

Hematoma requiring surgical drainage/exploration but no evidence of active bleeding

Rapidly expanding hematoma suggesting active bleeding requiring surgical exploration or referral

Mild

Moderate

Severe

CHM1

CHM2

CHM3

Infection Pain and erythema with no obvious swelling

Painful swelling with erythema or elevated temperature or purulent wound discharge

Cellulitis or wound necrosis

Mild

Moderate

Severe

CIN1

CIN2

CIN3

Device removal difficulties

Difficult removal, with pain score of 8 or more lasting for over 2 minutes

Difficult removal, with abrasion of shaft or glans

Difficult removal, requiring injection of local anesthetic or requiring up to three sutures post-removal

Difficult removal, requiring more than three sutures

Difficult removal, with penile damage

Mild

Mild

Mild

Moderate

Severe

CDR1.1

CDR1.2

CDR1.3

CDR2

CDR3

Damage to the penis

Mild bruising or abrasion, not requiring treatment

Bruise or abrasion to the glans or shaft of the penis requiring pressure dressing or surgery to control

Portion or all of the glans or shaft of the penis severed

Mild

Moderate

Severe

CDP1

CDP2

CDP3

D. Within 6 Weeks Post Removal

Pain Symptoms of pain requiring bed rest for less than half the day

Pain requiring bed rest for more than half day

Excruciating pain requiring total bed rest

Mild

Moderate

Severe

DPA1

DPA2

DPA3

Excessive bleeding More bleeding than usual, but easily controlled

Bleeding that requires suture to control

Blood transfusion or transfer to another facility for management required

Mild Moderate Severe

DBL1

DBL2

DBL3

Edema More edema than usual but not causing any discomfort to the patient

Moderate edema causing the patient discomfort, though managed with conservative measures

Severe edema, causing the patient discomfort, uncontrolled with conservative measures

Mild

Moderate

Severe

DED1

DED2

DED3

Hematoma Mild contained hematoma, not requiring any treatment

Hematoma requiring surgical drainage/exploration but no evidence of active bleeding

Rapidly expanding hematoma suggesting active bleeding requiring surgical exploration or referral

Mild

Moderate

Severe

DHM1

DHM2

DHM3

Infection Pain and erythema with no obvious swelling

Painful swelling with erythema or elevated temperature or purulent wound discharge

Cellulitis or wound necrosis

Mild

Moderate

Severe

DIN1

DIN2

DIN3

Damage to the penis

Mild bruising or abrasion, not requiring treatment

Bruise or abrasion to the glans or shaft of the penis requiring suture or surgery to control

Portion or all of the glans or shaft of the penis severed

Mild

Moderate

Severe

DDP1

DDP2

DDP3

Delayed wound healing

Healing takes longer than usual, but no extra treatment necessary

Additional non-operative treatment required

Requires re-operation to correct

Mild

Moderate

Severe

DDW1

DDW2

DDW3

Appearance When healing is complete, subject concerned, but no discernable deformity

When healing is complete, minimal deformity does not require re-operation

Significant deformity requires re-operation to correct

Mild

Moderate

Severe

DAP1

DAP2

DAP3

Problems with voiding

Transient complaint by subject that resolves without treatment

Requires a special return to the clinic, but no additional treatment

Requires referral to another facility for management

Mild Moderate Severe

DVO1

DVO2

DVO3

E. Six Weeks or More Post Removal

Infection Pain and erythema with no obvious swelling

Painful swelling with erythema or elevated temperature or purulent wound discharge

Cellulitis or wound necrosis

Mild

Moderate

Severe

EIN1

EIN2

EIN3

Delayed wound healing

Healing takes longer than usual, but no extra treatment necessary

Additional non-operative treatment required

Requires re-operation to correct

Mild

Moderate

Severe

EDW1

EDW2

EDW3

Appearance Subject concerned, but no discernable deformity

Minimal deformity does not require re-operation

Significant deformity requires re-operation to correct

Mild

Moderate

Severe

EAP1

EAP2

EAP3

Excessive skin removed

Client concerned, but there is no deformity on erection

Causes slight discomfort on erection but surgical correction not necessary

Interferes with sexual life and surgical correction is necessary

Mild

Moderate

Severe

EES1

EES2

EES3

Insufficient skin removed

Prepuce partially covers the glans only when extended

Prepuce still partially covers the glans and re-operation is required to correct

Not applicable

Mild

Moderate

Severe

EIS1

EIS2

EIS3

Torsion of penis Torsion is observable, but does not cause pain or discomfort

Causes mild pain or discomfort on erection, but additional operative work not necessary

Requires re-operation or transfer to another facility to correct the problem

Mild

Moderate

Severe

ETP1

ETP2

ETP3

Erectile dysfunction

Client reports occasional inability to have an erection

Client reports frequent inability to have an erection

Mild

Moderate

EED1

EED2

Client reports complete or near complete inability to have erections

Severe EED3

Psycho- behavioural problems

Client reports mild sexual dissatisfaction attributed to male circumcision, but no significant psycho-behavioral consequences

Client reports significant sexual dissatisfaction attributed to male circumcision, but no significant psycho-behavioral consequences

Significant depression or other psychological problems attributed by the participant to the male circumcision

Mild

Moderate

Severe

EPB1

EPB2

EPB3

Other AEs Other AEs are described below* --

Other AEs

• Liver or pancreatic abnormalities;

• Neurologic conditions of the central nervous system (e.g., meningitis, encephalitis, convulsions and headaches, visual and auditory disturbances, strokes), and peripheral neurologic conditions (e.g., peripheral neuropathies, motor weakness etc.);

• Myocarditis and pericarditis;

• Dermatologic condition affecting the genital, pubic or perianal areas or groin; and,

• Injuries and accidents.

Common illnesses unrelated to male circumcision will not be included in the “other AE” category to avoid unnecessary reporting of irrelevant events. Such AEs include the following:

• Malaria and other common parasitic infections;

• Gastrointestinal (GI) tract diseases, including diarrhea, gastroenteritis (bacterial, viral or parasitic), and other GI conditions, and oropharangeal infections;

• Respiratory illnesses (Upper Respiratory Tract Infections, acute lower respiratory infection, pleurisy), including tuberculosis, influenza or other respiratory infections;

• Angina, myocardial infarction, congestive cardiac failure;

• Dermatologic conditions (e.g., scabies, infected and uninfected rashes, pruritis, fungal infection), except those affecting the genital, pubic or perianal areas or groin

• Neoplasms not affecting the genitourinary tract; and,

• Surgical conditions not related to the intervention (e.g., hernias) and which do not require hospitalization.
