# Supplementary material for: Safety and efficacy of the PrePex device in HIV-positive men: A single-arm study in Zimbabwe
Source: PLoS One. 2017 Dec 8;12(12):e0189146. doi: 10.1371/journal.pone.0189146 (PMC5722373; doi:10.1371/journal.pone.0189146)
Supplement: S2 Text — (DOCX) [file pone.0189146.s003.docx]

HIV + STUDY ADVERSE EVENTS

Device-related Adverse Events

Participant 1: Self-removal

The participant was an informal vendor, 35 years of age and was of WHO clinical stage 1. His CD4 count on day of device placement was 497c/μl and was referred to the OI clinic since he was not on ART. The participant had a size E device applied on him on 26 October 2015 after all the study processes and procedures were met. On day 7 after device placement (2 November 2015), the participant did not turn up for the scheduled visit and so was followed up. It was discovered that the participant was in Mozambique and had removed the device on day 3 after device placement. On 3 November 2015, the client came at the site. On examination, swelling and necrotic of distal foreskin was noted. Debridement and secondary suturing was done. He was encouraged to come for daily G&I dressing. He was given Cloxacillin and Ibuprofen for pain. On the second day after suturing, the participant did not turn up for bandage removal. He was picked up from home and was seen by a doctor who ordered dressing with Betadine and was given Ciprofloxacin. After two days he came and bandage was removed and wound cleaned with Betadine solution. Client was instructed to do saline baths and to abstain from sexual intercourse. Complete wound healing was noted on day 37 after device placement. This event was reported to MRCZ.

Participant 2: Device Displacement

The participant, an unmarried man 39 years of age, had device size A applied on 2 November 2015. All the vital signs were fine on day of screening. He was of WHO clinical stage 2 and had a CD4 count of 172 c/μl. He was on first line of ARVs and was taking Tenolam E and Cotrimoxazole. He had been on ART for one year eleven months. Before device placement, he underwent a psychosocial interview and portrayed some good understanding of the procedure. The participant did not turn up for device removal on the scheduled day. On day 8, the participant was visited at home but he was not there. A message was left for him to visit the clinic. The participant was picked up from home on day 9 post placement. He said the device had been completely displaced on day 1 but had only noticed it the following morning. On examination the device displacement resulted in partial necrosis of the foreskin with gross oedema. There was no ulceration or evidence of sepsis noted. Dorsal slit circumcision was done and closed with 3/0 vicryl on a cutting, dressed with paraffin gauze. No antibiotics were given. Participant came for weekly review visits and seen to be completely healed on day 28. This event was reported to MRCZ.

Participant 3: Device Displacement

The participant, a married carpenter 39 years old, had size C device placed on 18 March 2016. All vital signs were normal on day of placement. He was on clinical stage 2 and had a CD4 count of 164 c/μl. He was on first line of ARVs and was taking Tenolam E. He had been on ART since 9 November 2009. The adverse event occurred while he was at home with the device on. The device had displaced on fifth day after placement. Participant said the device moved while scratching the penis which was itchy post placement. On examination of the site, foreskin was in full retraction and swollen distal to the device. Swelling of shaft and glands could be seen. No septic sores were noted. Surgical circumcision using the sleeve method was done. Sutured with 2/0 Vicryl and dressed with paraffin gauze. On day 7, the participant came for review. Sutureline was septic and graying in the frenular area. Swelling subsided, discolouring of the skin L 0.5cm between the ring line and swollen line (Black band) was noted. Participant was advised on cleaning the wound with salty water. Participant was given Ciprofloxacin and Brufen. On day 42, client was completely healed. This event was reported to MRCZ.

Participant 4: Device Displacement

The participant was a 32 year old married man who had size C device placed on 24 March 2016. On day of device placement, the participant had a CD4 count of 283 c/μl, was of WHO clinical stage 1 and was taking first line ARVs, Tenolam E and Cotrimoxazole. He had been initiated on ART on 20 March 2016. The adverse event occurred while the participant was at home with the device on. The participant came on the 28^th^ of March reporting that the device came off on third day after placement while taking a shower. On examination of the site, foreskin was in full retraction. Gross swelling of the penile shaft was noted but was not septic. Dorsal slit was done under local anaesthetic Lignocaine 2% and Bupivacaine 0.5%. Haemosthasis was achieved and suturing with 3.0 Vicryl done. Dressing of the wound was done and participant instructed to return on day 2 post operatively. Participant was given Ciprofloxacin and Brufen. On day 9 post device placement, participant came for review and bandage was removed and participant was encouraged to start salt baths. Sutures were intact. On day 12 post device placement, the wound area had no sutures and was septic. The doctor ordered daily G&I dressings and was given Cloxacillin 500mg. Participant was completely healed on day 52 post device placement. This event was reported to MRCZ.

Adverse events not related to the device

Participant 5:

The participant a married 45 years of age man was of WHO clinical stage 2 and on first line of ARVs taking Tenolam E. He had been initiated on ART on 30 October 2012. His CD4 count on day of device placement was 361 c/μl. The participant had size C device applied on 1 February 2016. The device was successfully removed on day 7 post placement and experienced pain level VAS 2 when the device was being removed. The participant came for all scheduled visits and did not report any pain. On day 37 post device placement, the participant came for a review visit but complained of difficulty in passing urine and was referred to the doctor. Upon further investigations, the problem had started in September 2015 before he came for circumcision. He had complete obstruction about 28 days post device placement. Upon realizing his problem, he had gone to Karanda Mission Hospital where catherisation was unsuccessful but urine started coming out. He was experiencing pain towards the meatal opening when passing urine. On examination the bladder was not full but had some suprapubic tenderness. USS done at a private hospital showed significant urine retention of about 59%. There were no stones seen. The diagnosis was urethral stricture with unknown immediate cause. An ultrasound was done and participant was referred to Parirenyatwa OPD urology. Participant was completely healed on day 49 post device placement. This event was reported to MRCZ.

Participant 6:

The participant was a 33 year old man who had a size C device applied on 4 April 2016 after all the study processes and procedures were met. On day of device placement, the participant had a CD4 count of 256 c/μl, was of WHO clinical stage 1 and was taking first line ARVs, Tenolam E. He had been on ART for about four and a half years. Participant healed on 9 May 2016, 35 days after device placement. The adverse event occurred while participant was killed in a road traffic accident at home on the 5^th^ of June 2016, nearly 60 days after circumcision. The study team only got to know about it on the 4^th^ of July after trying to contact the participant to remind him of the last review visit which had been scheduled for that day. This event was reported to MRCZ.
