# Supplementary material for: Safety and efficacy of the PrePex device in HIV-positive men: A single-arm study in Zimbabwe
Source: PLoS One. 2017 Dec 8;12(12):e0189146. doi: 10.1371/journal.pone.0189146 (PMC5722373; doi:10.1371/journal.pone.0189146)
Supplement: S3 Text — (DOCX) [file pone.0189146.s004.docx]

| **UNIVERSITY OF WASHINGTON**  **Human Subjects Division, Box 359470** |
| --- |
| **HUMAN SUBJECTS REVIEW COMMITTEE APPLICATION** |

**DO NOT SUBMIT THIS PAGE TO HSD**

**Do not complete this form if you are a member of the Cancer Consortium and your proposed research is cancer-related and not a repository.** Your research must be reviewed by the Cancer Consortium IRB administered by the Fred Hutchinson Cancer Research Center (FHCRC) IRB instead of the UW IRB. If you are unfamiliar with this process, see the Cancer Consortium website <http://www.cancerconsortium.org/en.html> or contact Cancer Consortium Clinical Research Support at 206-667-4520, or [CRScustomerservice@fhcrc.org](mailto:CRScustomerservice@fhcrc.org). For a list of Cancer Consortium members, see

<http://is-ext.fhcrc.org/sites/consortium/ccdb/members.php>.

**Do not complete this form if your research involves obtaining data from, or interacting with clients of, the Washington State Department of Health or Department of Social and Human Services.** Your research must be reviewed by the Washington State IRB instead of the UW IRB. If you are unfamiliar with this process, see the WA State IRB website

<http://www.dshs.wa.gov/rda/hrrs/> or contact their office at: 360-902-8075, or [wsirb@dshs.wa.gov](mailto:wsirb@dshs.wa.gov).

This project requires full IRB Committee Review.

**Collate into three complete packets:**

- Three copies of this form.
- Three copies of all relevant materials (advertisements, consent forms, data collection forms, debriefing statements, drug information summary, instruments, questionnaires, etc.)

**Add:**

- One full free-standing copy of each—Research Proposal (see specific instructions in Section VII for Center, Program, and Institutional Training grants); Grant or Contract; Protocol and Investigator’s Brochure (for clinical trials); Thesis or Dissertation Proposal (students only)

This project requires Minimal Risk (“Expedited”) Review. (See the documents called [SOP Expedited Review](http://www.washington.edu/research/hsd/docs/1640) and [WORKSHEET Expedited Review Eligibility](http://www.washington.edu/research/hsd/docs/1641) for information and a description of the eligibility requirements)

**Collate into two complete packets:**

- Two copies of this form.
- Two copies of all relevant materials (advertisements, consent forms, data collection forms, debriefing statements, drug information summary, instruments, questionnaires, etc.)

**Add:**

- One full free-standing copy of each—Research Proposal (see specific instructions in Section VII for Center, Program, and Institutional Training Grants ; Grant or Contract; Protocol and Investigator’s Brochure (for clinical trials); Thesis or Dissertation Proposal (students only)

*(Please note that it is ultimately HSD staff and IRB who make the review level determination.)*

**Send to:**

Human Subjects Division, Box 359470, Seattle, WA 98195.

When preparing double-sided copies, please make sure that each item (e.g., IRB application, consent form, questionnaires, etc.) begins on the front page of a new piece of paper.

Incomplete or handwritten forms are not accepted.

When completing the form, do not leave blanks, and use 10 point type or larger.

The contents of this application and attachments will be kept confidential within the limits of the law.

For more information visit the HSD website at <http://www.washington.edu/research/hsd/> or call (206) 543-0098.

|  | | **UNIVERSITY OF WASHINGTON** | BOX FOR COMMITTEE USE ONLY  MASTER  COMM.  INVESTIGATOR  |
| --- | --- | --- | --- |
|  | **Human Subjects Division**  **Box 359470**  **HUMAN SUBJECTS REVIEW COMMITTEE APPLICATION** | | APPLICATION NO. |

**I. PRINCIPAL INVESTIGATOR (Provide all the information requested. All paper-based correspondence will be directed to this person. Please list the mailing address for paper-based correspondence. You may designate a contact person other than yourself in section II., below.)**

| Name | **Scott Barnhart** | | | | | | | | | Title | | **MD MPH** | | | Position | | **Professor** | |
| --- | --- | --- | --- | --- | --- | --- | --- | --- | --- | --- | --- | --- | --- | --- | --- | --- | --- | --- |
| Home Institution (or source of paycheck)  **UW Student? Home institution is UW.** | | | | | | | | **University of Washington** | | | | | | | | | | |
| Home UW Department (if applicable) | | | | | | **Global Health** | | | | | | | | Division | I-TECH | | | |
| **UW Position or appointment (choose the most appropriate one):** | | | | | | | | | | | | | | | | | | |
|  | | | [x] Regular Faculty Appointment | | | | | | | | [ ] Research Faculty Appointment | | | | | [ ] Clinical Faculty Appointment | | |
| Faculty: | | | [ ] Visiting Faculty Appointment | | | | | | | | [ ] Dual Appointment with PNNL | | | | |  | | |
|  | | | [ ] Other (describe): | | | | | | | |  | | | | | | | |
| Student: | | | [ ] Matriculated Undergraduate Student | | | | | | | | [ ] Graduate or Professional Student (matriculated or approved “On Leave”) | | | | | | | [ ] WWAMI Student |
| [ ] Resident or Fellow at the UW or Local VA | | | | | | | | | | | | | [ ] UW Administration or Staff | | | | | [ ] None |
| Campus Box # | | | | **359932** | Other Address if not at UW | | | | | | | |  | | | | | |
| Telephone | | **206-685-4875** | | | | | Fax | | **206-221-4945** | | | | e-mail | | **sbht@uw.edu** | | | |

**II. CONTACT PERSON (Provide all the information requested. If this section is completed, all paper-based correspondence will be directed to this person.)**

| Name | **Melody Datz** | | | | | | Title | **MA** | | | Position | **Research & Publication Coordinator** |
| --- | --- | --- | --- | --- | --- | --- | --- | --- | --- | --- | --- | --- |
| Campus Box # | | | **359932** | Other Address if not at UW | | | | |  | | | |
| Telephone | | **206-543-1742** | | | Fax | **206-543-2464** | | | | e-mail | **medatz@uw.edu** | |

**III. TITLE OF PROJECT:** Evaluation of Safety, Efficacy and Acceptability of the PrePex™ Device for Rapid Scale up of Adult Male Circumcision Programs in Zimbabwe, Phase III PrePex™ field implementation trial with HIV positive adult males

**IV. SIGNATURES:** **The undersigned acknowledge that: 1. this application is an accurate and complete description of the proposed research; 2. the research will be conducted in compliance with the recommendations of and only after approval has been received from the Human Subjects Review Committee (HSRC). The lead research is responsible for all aspects of this research, including: reporting any serious adverse events or problems to the HSRC, requesting prior HSRC approval for modifications, and requesting continuing review and approval.**

| **A****. Investigator:** | **Scott Barnhart, MD, MPH** | |  |
| --- | --- | --- | --- |
|  | | **Typed name plus signature** | **DATE** |
| **B****. Faculty sponsor (for student):** |  | |  |
|  | | **Typed name plus signature** | **DATE** |
| **C. The Chair, Dean, or Director acknowledges the researcher is qualified to do the research, sufficient resources will be available, and (if no external funding review occurred) there was an internal review of scientific merit.** | | | |
|  | **Ann Downer, EdD, MS** | |  |
|  | | **Typed name plus signature** | **DATE** |

__________________________________________________________ _____________ APPROVE  DISAPPROVE 
 HUMAN SUBJECTS REVIEW Committee signature Date

Subject to the following restrictions: _____________________________________________________________________________

______________________________________________________________________________________________________

Period of approval is one year, from_____________________ through________________________

 Subject numbers are approved as described in this Human Subjects Review Application unless otherwise indicated above in “Subject to the following conditions” or in an accompanying letter.

***VALID ONLY AS LONG AS APPROVED PROCEDURES ARE FOLLOWED***

| **V. TYPE OF NEW SUBMISSION** | | | | | | | | | |
| --- | --- | --- | --- | --- | --- | --- | --- | --- | --- |
| **[]** | **MINIMAL RISK**  (The research meets the **definition of minimal risk** and falls into one or more [**expedited review categories**](http://www.washington.edu/research/hsd/docs/1641)**. You are done with this table.**) | | | | | | | | |
| **[ X ]** | **FULL COMMITTEE** | | | | | | | | |
|  | **[ X ]** | Full Committee (The research involves **greater than minimal risk** and requires review at a convened meeting of the IRB) | | | | | | | |
|  | **[ ]** | Full Committee (The research involves **no more than minimal risk** but does not fit into one of the [expedited review categories)](http://www.washington.edu/research/hsd/docs/1641) | | | | | | | |
|  | **[ ]** | **METHODS:** | | | | | | | |
|  |  | Please mark the boxes to indicate the methods which best describe your study: | | | | | | | |
|  |  | **[ ]** | | **Social-Behavioral Procedures/Considerations** | | | | | |
|  |  |  | **[X ]** | | | Observational | | **[ ]** | Population-Based Field Study |
|  |  |  | **[ ]** | | | Behavioral Interventions | | **[ ]** | Surveys/Questionnaires |
|  |  |  | **[ X ]** | | | Interview/Focus Groups | | **[ ]** | Other – Describe: |
|  |  | **[ ]** | **Medical Procedures/Considerations** | | | | | | |
|  |  |  | **[ ]** | | Bio-hazardous Substances | | **[ X ]** | | Investigational/Approved Devices |
|  |  |  | **[ ]** | | Controlled Substances | | **[ ]** | | Radiation Exposure |
|  |  |  | **[ ]** | | Emergency Treatment | | **[ ]** | | Substance Abuse Treatment (with medication) |
|  |  |  | **[ ]** | | Gene Transfer Study | | **[ ]** | | Surgical Procedures |
|  |  |  | **[ ]** | | Stem Cell Research | | **[ ]** | | Genetic Testing |
|  |  |  | **[ ]** | | Magnetic Resonance imaging (MRI) | | **[ ]** | | Complementary/Alternative Medicine |
|  |  |  | **[ ]** | | Investigational/Approved Drugs and Biologics | | **[ ]** | | Other – Describe: |
|  | **[ ]** | **DOES YOUR RESEARCH INVOLVE OR IS IT ASSOCIATED WITH ANY OF THE FOLLOWING:** | | | | | | | |
|  |  | **[ ]** | Emergency Medicine | | | | | | |
|  |  | **[ ]** | Pregnant Women as a Target Population | | | | | | |
|  |  | **[ ]** | Genetics | | | | | | |
|  |  | **[ ]** | Stem Cells | | | | | | |
|  |  | **[ ]** | Neuroscience | | | | | | |
|  |  | **[ ]** | College of Arts and Sciences | | | | | | |
|  |  | **[ ]** | College of Education | | | | | | |
|  |  | **[ ]** | Dentistry | | | | | | |
|  |  | **[ ]** | Infectious Disease | | | | | | |
|  |  | **[x ]** | HIV/AIDS | | | | | | |
|  |  | **[ ]** | Psychiatry | | | | | | |
|  |  | **[ ]** | Rehabilitation Medicine | | | | | | |
|  |  | **[ ]** | Psycho-Social Drug Abuse Research | | | | | | |
|  |  | **[ ]** | Alaska Native/American Indian (ANAI) | | | | | | |
|  |  | **[ ]** | Public Health | | | | | | |
|  |  | **[ x ]** | Global Health | | | | | | |
|  |  | **[ x ]** | Health Services | | | | | | |
|  |  | **[ x ]** | Quality of Care / Quality of Life | | | | | | |
|  |  | **[ ]** | Health Prevention / Health Education | | | | | | |
|  |  | **[ ]** | Nursing | | | | | | |

**VI. CO-INVESTIGATORS (Provide all the information requested for each co-investigator. Add sheets if necessary.)**

| Name | Mufata Tshimanga | | | | | | Title | MD | | | | Position | | ZiCHIRe Executive Director |
| --- | --- | --- | --- | --- | --- | --- | --- | --- | --- | --- | --- | --- | --- | --- |
| Department | | |  | | | | | | Division |  | | | | |
| Mail box or address | | | | 28 Van Praagh Ave, Milton Park, Harare | | | | | | | | | | |
| Telephone | | (263 4) 762165 | | | Fax |  | | | | | e-mail | | tshimangamufuta@gmail.com | |
| Name | | Gerald Gwingi | | | Title | MBChB, MPH, MSc | | | | | Position | | Permanent Secretary | |
| Department | | | Zimbabwe Ministry of Health and Child Care | | | | | | Division |  | | | | |
| Mail box or address | | | | Cnr 4^th^ Street/Central Ave, PO Box CY1122, Harare, Zimbabwe | | | | | | | | | | |
| Telephone | | (263 4) 798573 | | | Fax |  | | | | | e-mail | ggwinji@gmail.com | | |

| Name | Batsirai Makunike | | | | | | Title | MD, MPH | | | | Position | Country Director, I-TECH/Zimbabwe |
| --- | --- | --- | --- | --- | --- | --- | --- | --- | --- | --- | --- | --- | --- |
| Department | | | Global Health | | | | | | Division | I-TECH | | | |
| Mail box or address | | | | 15 Phillips Ave, Belgravia, Harare, Zimbabwe | | | | | | | | | |
| Telephone | | (263 4) 704890 | | | Fax |  | | | | | e-mail | bmakunike@itech-zimbabwe.org | |

| Name | Caryl Feldacker | | | | | | Title | PhD, MPH | | | | Position | Research & Evaluation Advisor |
| --- | --- | --- | --- | --- | --- | --- | --- | --- | --- | --- | --- | --- | --- |
| Department | | | Global Health | | | | | | Division | I-TECH | | | |
| Mail box or address | | | | 359932 | | | | | | | | | |
| Telephone | | 206-543-3456 | | | Fax |  | | | | | e-mail | cfeld@uw.edu | |

| Name | Owen Mugurungi | | | | | | Title | MD | | | | Position | Director |
| --- | --- | --- | --- | --- | --- | --- | --- | --- | --- | --- | --- | --- | --- |
| Department | | | Zimbabwe Ministry of Health and Child Care | | | | | | Division | HIV and TB Programme | | | |
| Mail box or address | | | | 10 4th Street, Marondera | | | | | | | | | |
| Telephone | | (263) 792 4760 | | | Fax |  | | | | | e-mail | nacp@telco.co.zw | |

| Name | Taurayi Mangwiro | | | | | | Title | MBChB, MMed | | | | Position | Urologist |
| --- | --- | --- | --- | --- | --- | --- | --- | --- | --- | --- | --- | --- | --- |
| Department | | | Department of Surgery | | | | | | Division | University of Zimbabwe | | | |
| Mail box or address | | | | Suite 10 2nd Floor Fife Avenue Medical Centre, Fife Avenue, Harare | | | | | | | | | |
| Telephone | | (263) 772 544 380 | | | Fax |  | | | | | e-mail | timangwiro@yahoo.co.uk | |

| Name | Peter Kilmarx | | | | | | Title | MD | | | | Position | Country Director |
| --- | --- | --- | --- | --- | --- | --- | --- | --- | --- | --- | --- | --- | --- |
| Department | | | Centers for Disease Control and Prevention | | | | | | Division | Zimbabwe Country Office | | | |
| Mail box or address | | | | 38 Samora Machel Avenue; Harare, Zimbabwe | | | | | | | | | |
| Telephone | | (263 4) 796-040 | | | Fax |  | | | | | e-mail | pbk4@cdc.gov | |

| Name | Sinokuthemba Xaba | | | | | | Title | RGN, BSc, MSc | | | | Position | MC & Condom Coordinator |
| --- | --- | --- | --- | --- | --- | --- | --- | --- | --- | --- | --- | --- | --- |
| Department | | | Zimbabwe Ministry of Health and Child Care | | | | | | Division | HIV and TB Programme | | | |
| Mail box or address | | | | 10 4th Street, Marondera | | | | | | | | | |
| Telephone | | 0776 072 731 | | | Fax |  | | | | | e-mail | xabasino@gmail.com | |

| Name | Danuta Kasprzyk | | | | | | Title | PhD | | | | Position | Research Scientist |
| --- | --- | --- | --- | --- | --- | --- | --- | --- | --- | --- | --- | --- | --- |
| Department | | | Battelle Memorial Institute | | | | | | Division | Centers for Public Health Research & Evaluation | | | |
| Mail box or address | | | | 1100 Dexter Avenue N, Seattle, WA 98109 | | | | | | | | | |
| Telephone | | 206-528-3106 | | | Fax |  | | | | | e-mail | kasprzyk@battelle.org | |

| Name | Munyaradzi Murwira | | | | | | Title | MBChB, MPH | | | | Position | Executive Director |
| --- | --- | --- | --- | --- | --- | --- | --- | --- | --- | --- | --- | --- | --- |
| Department | | | Zimbabwe National Family Planning Council | | | | | | Division |  | | | |
| Mail box or address | | | | P.O. Box ST220 Southerton, Harare | | | | | | | | | |
| Telephone | | (263 4) 620 281 | | | Fax |  | | | | | e-mail | ed@znfpc.org.zw | |

| Name | Amy Herman-Roloff | | | | | | Title | Ph.D., M.P.H. | | | | Position | Chief Science Officer |
| --- | --- | --- | --- | --- | --- | --- | --- | --- | --- | --- | --- | --- | --- |
| Department | | | Centers for Disease Control and Prevention | | | | | | Division | Zimbabwe Country Office | | | |
| Mail box or address | | | | 38 Samora Machel Avenue; Harare, Zimbabwe | | | | | | | | | |
| Telephone | | **263-772-161-718** | | | Fax |  | | | | | e-mail | bjy2@cdc.gov | |

| Name | Stephanie Marie Davis | | | | | | Title | MD, MPH | | | | Position | Medical Epidemiologist |
| --- | --- | --- | --- | --- | --- | --- | --- | --- | --- | --- | --- | --- | --- |
| Department | | | Centers for Disease Control and Prevention | | | | | | Division | Division of Global HIV/AIDS | | | |
| Mail box or address | | | | 1600 Clifton Rd, Mail Stop E-04 | | | | | | | | | |
| Telephone | | (404) 639-6340 | | | Fax |  | | | | | e-mail | smdavis@cdc.gov | |

| Name | Jason Reed | | | | | | Title | MD, MPH | | | | Position | Senior Technical Advisor |
| --- | --- | --- | --- | --- | --- | --- | --- | --- | --- | --- | --- | --- | --- |
| Department | | | Office of the U.S. Global AIDS Coordinator | | | | | | Division |  | | | |
| Mail box or address | | | | SA-29, 2nd floor 2201 C. Street NW Washington, DC 20522-2920 | | | | | | | | | |
| Telephone | | 202-663-2440 | | | Fax |  | | | | | e-mail | reedjb@state.gov | |

| Name | Panganai Dhliwayo | | | | | | Title | MBChB, MPH | | | | Position | Deputy Director for Programs |
| --- | --- | --- | --- | --- | --- | --- | --- | --- | --- | --- | --- | --- | --- |
| Department | | | Centers for Disease Control and Prevention | | | | | | Division | Zimbabwe Country Office | | | |
| Mail box or address | | | | 38 Samora Machel Avenue; Harare, Zimbabwe | | | | | | | | | |
| Telephone | | 263 796040 | | | Fax |  | | | | | e-mail | vhr0@cdc.gov | |

**VII. SECTION 1 - List each proposed and funded grant or contract** **relevant to this application, AND ATTACH A COMPLETE COPY OF EACH GRANT OR CONTRACT. THIS SHOULD INCLUDE GRANTS THAT SUPPORT FACULTY TIME FOR DATA ANALYSIS AND MANUSCRIPT PREPARATION, (I.E. SALARY SUPPORT). IF NONE, CHECK HERE** **. FOR CENTER OR PROGRAM PROJECT GRANTS LIST P.I. AND TITLE FOR EACH SEPARATE PROJECT OR CORE. aDD sheetS if necessary.**

For Center, Program, and Institutional Training Grants (e.g., NIH “P” awards and “T” awards): Attach only the following components of the application. The terms used here are from the standard NIH applications for these types of grants. If the grant is from another agency, provide the equivalent application sections.

- Cover page(s)
- Project/Performance Site Location
- Other Project Information
- Research Plan (for Center or Program grants)
- Research Training Program Plan (for Institutional Training grants)
- Biosketch (profile) for the principal investigator on the grant

| A. Type of proposal: | | |  | | | Research | | | | |  | | Contract | | | |  | | | Fellowship | | | |  | | Training grant | | |  | Subcontract |  |  |
| --- | --- | --- | --- | --- | --- | --- | --- | --- | --- | --- | --- | --- | --- | --- | --- | --- | --- | --- | --- | --- | --- | --- | --- | --- | --- | --- | --- | --- | --- | --- | --- | --- |
| Other, specify | | | | **Cooperative Agreement** | | | | | | | | | | | | | | | | | | | | | | | | | | | | |
|  | | | | | | | | | | | | | | | | | | |  | | | | | | | | | | | | |  |
| B. Name of principal investigator: | | | | | | | | | | **Scott Barnhart** | | | | | | | | | | | | | | | | | | | | | |  |
| C. Name of funding agency: | | | | | | | **Center for Disease Control and Prevention (CDC)** | | | | | | | | | | | | | | | | | | | | | | | | |  |
| D. Agency's number (if assigned): | | | | | | | | | | **Cooperative Agreement grant number: 5U2GGH000972-02 REVISED (Appendix 12)** | | | | | | | | | | | | | | | | | | | | | |  |
| E. Title of proposal: | | | **Overall Co-AG name: Scaling up voluntary male circumcision to prevent HIV transmission in Zimbabwe (Appendix 11)**  **Proposal name: Evaluation of Safety, Efficacy and Acceptability of the PrePex**™ **Device for Rapid Scale up of Adult Male Circumcision Programs in Zimbabwe, Phase III PrePex**™ **field implementation trial with HIV positive adult males** | | | | | | | | | | | | | | | | | | | | | | | | | | | | |  |
| F. Inclusive dates: from | | | | | **10/15/2014** | | | | | | | through | | | | **4/14/2016** | | | | | | |  |  |  |  |  |  |  |  |  |  |
| G. Status: |  | New | | |  | | | Competing renewal | | | | | | |  | | | Non-competing renewal | | | | | | | | | |  |  |  |  |  |
|  | | | | | | | | |  | | | | |  | | | | | | | |  | | | | |  |  |  |  |  |  |
| H. Submitted through UW Office of Sponsored Programs? | | | | | | | | | | | | | | | | | |  | | | Yes |  | | | No, (attach explanation) | | | | | | | |

**---------------------------------------------------------------------------------------------------------------------------**

**---------------------------------------------------------------------------------------------------------------------------**

| A. Type of proposal: | | | |  | | | Research | | | | |  | | Contract | | | |  | | | Fellowship | | | | | |  | | Training grant | | |  | Subcontract |  |  |
| --- | --- | --- | --- | --- | --- | --- | --- | --- | --- | --- | --- | --- | --- | --- | --- | --- | --- | --- | --- | --- | --- | --- | --- | --- | --- | --- | --- | --- | --- | --- | --- | --- | --- | --- | --- |
|  | |  | | | | | | | | | | | | | | | | | | | | | | | | | | | | | | | | |  |
| Other, specify | | | | |  | | | | | | | | | | | | | | | | | | | | | | | | | | | | | | |
|  | | | | | | | | | | | | | | | | | | | |  | | | | | | | | | | | | | | |  |
| B. Name of principal investigator: | | | | | | | | | | |  | | | | | | | | | | | | | | | | | | | | | | | |  |
| C. Name of funding agency: | | | | | | | |  | | | | | | | | | | | | | | | | | | | | | | | | | | |  |
| D. Agency's number (if assigned): | | | | | | | | | | |  | | | | | | | | | | | | | | | | | | | | | | | |  |
| E. Title of proposal: | | | |  | | | | | | | | | | | | | | | | | | | | | | | | | | | | | | |  |
| F. Inclusive dates: from | | | | | |  | | | | | | | through | | | |  | | | | | | | | |  |  |  |  |  |  |  |  |  |  |
|  | | | | | | | | | |  | | | | |  | | | | | | | |  | | | | | | |  |  |  |  |  |  |
| G. Status: |  | | New | | |  | | | Competing renewal | | | | | | |  | | | Non-competing renewal | | | | | | | | | | | |  |  |  |  |  |
|  | | | | | | | | | |  | | | | |  | | | | | | | | |  | | | | | |  |  |  |  |  |  |
| H. Submitted through UW Office of Sponsored Programs? | | | | | | | | | | | | | | | | | | |  | | | Yes | | |  | | | No, (attach explanation) | | | | | | | |

**---------------------------------------------------------------------------------------------------------------------------**

| A. Type of proposal: | | | |  | | | Research | | | | |  | | Contract | | | |  | | | Fellowship | | | | |  | | Training grant | | |  | Subcontract |  |  |
| --- | --- | --- | --- | --- | --- | --- | --- | --- | --- | --- | --- | --- | --- | --- | --- | --- | --- | --- | --- | --- | --- | --- | --- | --- | --- | --- | --- | --- | --- | --- | --- | --- | --- | --- |
|  | |  | | | | | | | | | | | | | | | | | | | | | | | | | | | | | | | |  |
| Other, specify | | | | |  | | | | | | | | | | | | | | | | | | | | | | | | | | | | | |
|  | | | | | | | | | | | | | | | | | | | |  | | | | | | | | | | | | | |  |
| B. Name of principal investigator: | | | | | | | | | | |  | | | | | | | | | | | | | | | | | | | | | | |  |
| C. Name of funding agency: | | | | | | | |  | | | | | | | | | | | | | | | | | | | | | | | | | |  |
| D. Agency's number (if assigned): | | | | | | | | | | |  | | | | | | | | | | | | | | | | | | | | | | |  |
| E. Title of proposal: | | | |  | | | | | | | | | | | | | | | | | | | | | | | | | | | | | |  |
| F. Inclusive dates: from | | | | | |  | | | | | | | through | | | |  | | | | | | | |  |  |  |  |  |  |  |  |  |  |
|  | | | | | | | | | |  | | | | |  | | | | | | | |  | | | | | |  |  |  |  |  |  |
| G. Status: |  | | New | | |  | | | Competing renewal | | | | | | |  | | | Non-competing renewal | | | | | | | | | | |  |  |  |  |  |
| H. Submitted through UW Office of Sponsored Programs? | | | | | | | | | | | | | | | | | | |  | | | Yes | |  | | | No, (attach explanation) | | | | | | | |

**---------------------------------------------------------------------------------------------------------------------------**

**VIII. SECTION 2 - TRAINING VERIFICATION & REPORT**

1. Certification of training in the protection of human research subjects should be attached to this application if:
2. Funding was indicated in VI. Section 1 from any of the following sources:
   1. the National Institutes of Health (NIH);
   2. the Department of Defense (DoD) or a DoD component (e.g. Department of the Navy (DoN), Air Force, etc.);

*[NOTE – Complete the* [*Supplement: Department of Defense Involvement*](http://www.washington.edu/research/hsd/docs/542)*. ]*

- 1. a sponsor for which completion of training in the protection of human research subjects is a requirement to receive funding.

**OR**

1. The study is being conducted in collaboration with a non-UW institution/organization, including DoD involvement, that requires the completion of training in the protection of human research subjects (e.g., FHCRC, VA, DoD, etc.).

The [Certification of Training](https://www.washington.edu/research/hsd/verify/) is available on the HSD website (<http://www.washington.edu/research/hsd/verify/>). The Principal Investigator/Lead Researcher and all who are considered to be “senior/key personnel” by NIH, or who are required to complete training under DoD or another sponsor’s requirements, should be listed on the same Certification. Search first by the PI’s last name and select by clicking on the correct record. List subsequent individuals in the same order they are listed on the grant application. Click on “View Report” to generate a PDF of the Certificate of Training; print the report; and include it with the IRB application.

1. Does the sponsor or collaborating institution/organization require “refresher/renewal” training in the protection of human research subjects? No Yes If yes, please state how often (e.g. every three years):

**IX. SUMMARY OF ACTIVITY**. Answer in spaces provided (add numbered, referenced, single-sided sheets when necessary). Do not refer to an accompanying grant or contract proposal.

**The Primary Objectives are**:

- 1. To assess the safety of the PrePex™ device when applied to HIV positive men by assessing the rate of the clinical adverse events.

1. **The Secondary Objectives are:**
   1. To assess the level of care required to provide PrePex™ MC to HIV positive men, when taking into account the following parameters:
      1. PrePex™ procedure duration (a combination of the application and removal procedure times)
      2. Pain management at different stages of the procedure
      3. Time to complete healing, defined as, intact epithelium (unbroken skin) covering the epithelial as judged by the provider on visual inspection, meaning that none of the following are present: sutures, scabbing, drainage, moisture, gaps between epithelial edges or ulceration.]
   2. To evaluate the acceptability of the PrePex™ procedure to HIV-positive men as well as service providers.
   3. To evaluate Facilitators/Barriers for seeking VMMC among HIV-infected men
   4. To assess compliance with instructions to refrain from sexual intercourse until seven weeks post-placement-- Day 49 post-placement visit (6 weeks post-removal) or as instructed by a clinician, whichever is later, as a public health safety issue.
   5. Assess AE monitoring and reporting systems with respect to completeness and timeliness of reporting

**Intended use of findings**

The findings of the study will first and foremost provide further evidence about the safety of PrePex™ when used in HIV-positive men in resource limited settings and can be used to inform the roll-out of VMMC services for HIV-positive men using the PrePex™ device in Zimbabwe. The findings will also be used to make recommendations regarding training of clinicians and appropriate messages to be conveyed to HIV positive participants in pre-procedure counseling sessions.

Ultimately these findings will contribute to the body of knowledge required by WHO for the prequalification of PrePex™ for use in HIV positive men in Zimbabwe. We also intend to disseminate these findings widely through presentations at international meetings and publications in international peer-reviewed journals.

**A. BACKGROUND AND PURPOSE OF RESEARCH.** Provide relevant background information and explain **in lay language** why this research is important and what question(s) or hypotheses this activity is designed to answer.

The Zimbabwean Ministry of Health and Child Care (MOHCC), with assistance from the International Training and Education Center for Health (I-TECH), Department of Global Health, University of Washington and our partners, the Zimbabwe Association of Church-Related Hospitals (ZACH) and the Zimbabwe Community Health Intervention Project (ZiCHIRe), are training health care workers and supporting roll-out of adult voluntary medical male circumcision (VMMC) services. Two techniques for VMMC are currently approved for use in Zimbabwe, the forceps-guided method, which is a surgical technique, and PrePex™ method, which uses a device to perform the procedure. This IRB application is for I-TECH’s effort to support the MOHCC with data on adverse events rates when employing the PrePex™ device among HIV+ men, a group of men previously considered outside of the target group for the national VMMC program. Information from this study will help the MOHCC decide whether they should mandate an HIV test prior to performing male circumcision on a client by providing safety information for PrePex™ among HIV+ men. If HIV+ men do not have higher rates of AEs than HIV- men, HIV testing may no longer be mandatory, removing a barrier to uptake of VMMC.

**Background**

According to mathematical modeling, Voluntary Medical Male Circumcision (VMMC) has the potential to have a major impact on reducing HIV incidence in areas with high HIV prevalence and low prevalence of male circumcision (MC), as is the case in Zimbabwe. Recent estimates have shown that 42% or nearly 600 000 new infections could be averted in Zimbabwe between 2011 and 2025 if 80% of men and adolescents aged 15 -49 would be circumcised between 2012 and 2016 [[1](#_ENREF_1)] . MC is offered by Ministry of Health and Child Care (MOHCC) as part of a standard, minimum package of integrated HIV prevention services comprised of Testing and Counseling, screening and treatment for sexually transmitted infections, counseling on safer sexual practices, female and male condoms, as well as a safe MC procedure using the forceps-guided surgical method. From 2009 to date nearly 155 000 male circumcisions have been conducted in Zimbabwe using forceps-guided method. At the rate of 30-40,000 MCs/year, it might take up to 30 years to reach the target of circumcising 1.2 million men. Innovative strategies are therefore required to scale up MC services in Zimbabwe.

Male circumcision devices have the potential to accelerate VMMC programme scale up as the procedure seems to be faster and simpler and can be safely performed by nurses [[2](#_ENREF_2)] . Devices may also be more acceptable than surgery as they do not cause much pain and do not interrupt activities of daily living [[2](#_ENREF_2)] . Devices which facilitate surgical MC are widely used in infants and young boys, but experience in adolescents and HIV positive adults with devices that replace surgery is limited, particularly in countries in the African region where rapid expansion of male circumcision programmes for HIV prevention is most urgent.

Zimbabwe has piloted the PrePex device in a series of clinical trials since October 2011. The PrePex™ MC device was developed and manufactured in Israel by CircMedTech. It does not require local anaesthesia and is bloodless. The device’s circumcision mechanism is based on controlled necrosis of the foreskin over a one-week period. The PrePex**^TM^** device was assessed in Zimbabwe in a three phase trial which included clinical and psychosocial objectives. The three phase clinical trial of the “PrePexTM” Device for use in scaling up male circumcision (MC) in Zimbabwe started on 24th October, 2011 at Spilhaus centre in Harare. Studies in Phase I and Phase II of the trial (Protocol # MRCZ/A/1628), have established the safety and efficacy of PrePexTM among HIV-negative men, and the ongoing Phase III trial is trying to establish the safety and efficacy of task shifting PrePex circumcision from doctors to nurses for those populations. The previous trials included: 1) a Safety phase to determine the safety of the PrePex**^TM^** device among HIV+ men; 2) a Comparative phase to assess its performance compared directly to surgical MCs; and 3) a Field Study phase to assess the ease of use among trained nurses or general practitioners in an implementation roll out in District Hospitals rather than at the centrally located MC Centre. The findings from these three trials have since established the safety and efficacy of the PrePex™ device, and allowed Zimbabwe’s MOHCC to present reports to WHO for pre-qualification approval of the PrePex™ device. Currently, the WHO has pre-qualified PrePex for HIV-negative men. Zimbabwe has since obtained WHO pre-qualification to use the PrePex™ device for circumcision of HIV-negative adult men.

In preparation for massive MC roll-out, the Ministry of Health and Child Care plans to further pilot PrePex™ circumcisions in different health care settings and special populations in a Phase III trial by performing 10,000 PrePex MCs in different settings and populations, including boys 13-17 and HIV+ men, in preparation for mass adult male circumcision. In this regard, MOHCC commissioned in 2012 an additional safety trial of PrePex Circumcision Device when performed by nurses in adolescent males aged 13-17 years and has further identified key operational research questions to determine how best to implement both device and surgical circumcisions in the national roll-out programme. One of these questions is: Can MC using the PrePex device be safely provided to other special populations, such as HIV-positive adult male participants? Prior studies of VMMC using PrePex™ in Zimbabwe have shown rates of AE ranging from 0.7 % to 3.8% [[2](#_ENREF_2)] . The most recent large-scale trial using nurses to provide the procedure in Zimbabwe had an AE rate of 0.7%. These trials were among HIV-negative men; the trials were conducted by the research team included in this IRB application. The Zimbabwe IRB approval for the previous study of HIV-negative men in 2012 and 2013 are attached in Appendices 13 and 14.

A major barrier to the scale up of VMMC may be the potential stigma associated with being identified as HIV+ [[3](#_ENREF_3)] [[4](#_ENREF_4)]. This may translate to men avoiding VMMC because of the requirement of obtaining an HIV test [[5](#_ENREF_5)] [[6](#_ENREF_6), [7](#_ENREF_7)] before VMMC. If PrePex™ is shown to be safe among HIV+ men then the need to require mandatory HIV testing greatly lessens and one would argue strongly to eliminate mandatory testing as this is a major barrier to VMMC. In addition to avoiding stigmatization, HIV-positive men may seek VMMC for other health benefits [[8](#_ENREF_8)] , such as reduced risk of sexually transmitted infections (STI) for men (genital ulcer disease and human papillomavirus infection) and their female partners (such as *Trichomonas vaginalis*, bacterial vaginosis, *HSV-2*, and human papillomavirus (the virus that causes cervical cancer), possible decreased risk of HIV-1 transmission to female partners [[9](#_ENREF_9)] , for improved and ease of hygiene, or to conceal their HIV infection status as increased members of HIV negative men undergo VMMC. As VMMC services scale up, increasing numbers of men with unknown HIV infection status will seek VMMC, some of whom will be undiagnosed HIV-positive. Consequently, the current practice of excluding HIV-infected men in VMMC programs could worsen stigmatization and to avoid indirect identification, HIV positive men might seek surgery from potentially unsafe sources to mask their sero-status; and circumcised HIV-uninfected men might use their circumcision status to negotiate unsafe sex. Therefore, the safety of use of PrePex™ for VMMC of HIV-infected men must be established.

## Public Health Relevance of Proposed Study

As discussed above, stigma and desire to avoid HIV testing remain potential barriers. If PrePex can be used safely among all men, without determining the client’s HIV status, then the barrier of mandatory testing can be removed. Determining PrePex safety in HIV+ men would also allow both those with unknown status and those with positive status to undergo MC with PrePex. Reviewed below are the recent studies on VMMC among HIV+ men including rates of AE and wound healing time among those with CD4 greater or less than 350.

### **Differences in AE rates between HIV+ and HIV- individuals**

A few studies have been completed to assess the differences in AE rates between HIV+ and HIV- individuals. One large study has been conducted in Rakai Uganda that included HIV+ and HIV- men. It used the sleeve circumcision procedure and only enrolled HIV+ men with a CD4 count greater than 350. It found no difference in the AE rate comparing HIV+ to HIV- men overall, but did find that HIV+ men had a higher probability of having wound dehiscence [[10](#_ENREF_10)]. A second study conducted in Kenya also found no association, but this was a secondary analysis and was underpowered [[11](#_ENREF_11)] .

Details:

The Ugandan study enrolled 2,326 HIV- and 420 HIV+ men. It included follow-up visits with active surveillance: visits were done at 24-48 hours, 5-9 days and 4-6 weeks with over 90% of men attended the 4-6 week visit. 6% of HIV+ men and 7.4% of HIV- men experienced at least one AE. Including only moderate and severe AEs, 3.1% of HIV+ men and 3.5% of HIV- men had a moderate or severe AE. Wound dehiscence was more common among HIV+ than HIV- men (2.6% to 1.3%) although they did not adjust for multiple comparisons (p=0.04). They calculated adjusted odds ratios to control for possible confounders, and found that HIV+ men had a 21% reduction in the odds of any AE and a 9% reduction in the odds of a moderate or severe AE compared to HIV- men [[10](#_ENREF_10)]. In a recent follow-up study of a subset of men from the same study to determine whether it was necessary to collect CD4 prior to MC, among 242 HIV + men , AE rates of 0.79 for those with CD4 >350, 1.19 for those with CD4 < 350 (p=.214) [[12](#_ENREF_12)]. They determined that pre-surgical CD4 count was unnecessary for safety.

The second study [[11](#_ENREF_11)] used the forceps-guided method, was conducted in Kenya, and had two arms: one with passive surveillance and one with active surveillance. Their main comparison was AE rates in the active and passive surveillance groups, but they enrolled a total of 119 HIV+ men across the two arms, so they did a secondary analysis of AE rates across HIV-status. They found that in the passive surveillance group, HIV+ men had 1.3-times (95%CI: 0.41-4-4) the odds of an AE while in the active surveillance group those who were HIV+ had 0.61 (0.15-2.6) times the odds of an AE. Since this was an underpowered secondary analysis with small sample sizes of HIV+ men, the odds rations have large confidence intervals and little can be concluded from this publication [[11](#_ENREF_11)] .

In summary, there is growing evidence to support the safety of surgical VMMC in HIV+ men; including those at more advanced stages of disease, however this has not been confirmed with the PrePex device.

### **MC benefits for HIV+ men and their partners**

Male circumcision has been shown to reduce the risk of acquiring other STIs besides HIV. It is well established that MC is associated with reduced prevalence of HPV infection among HIV- individuals, and there is evidence linking MC with decreased HPV incidence among both HIV- and HIV+ individuals [[13](#_ENREF_13)] [[14](#_ENREF_14)] [[15](#_ENREF_15)] . A study of HIV+ men failed to find an association between MC and reduced HPV transmission to their female partners [[16](#_ENREF_16)] , although female partners might still benefit indirectly from their partners’ reduced risk of HPV acquisition. A large RCT involving 5,534 HIV-negative men with 24 months of follow-up found that male circumcision was associated with reduced risk of incident HPV and HSV-2, but not syphilis [[17](#_ENREF_17)] .

Details:

A meta-analysis of observational studies [[13](#_ENREF_13)] found that circumcised men were less likely to have prevalent genital HPV infection that uncircumcised men (OR = 0.57, 95% CI 0.45-0.71). It also found “weak evidence” that circumcision was associated with decreased HPV incidence (RR = 0.75, 95% CI 0.37-0.60). These studies were among HIV- men.

In a Ugandan study among HIV+ men, the researchers found that MC significantly reduced the incidence and prevalence of multiple high-risk-HPV and low-risk- HPV infections. (RR = 0.53, 95% CI, 0.33-0.83) [[14](#_ENREF_14)]

An RCT in South Africa found that the prevalence of high-risk HPV among men who received MC was 14.8% (94/637) compared to 22.3% (140/627) among controls, with a PRR of 0.66 (95% CI 0.51-0.86). This was the first RCT to show a reduction in the prevalence of urethral high-risk HPV infection after MC [[15](#_ENREF_15)] .

A study in Uganda tested female partners of HIV+ men with CD4 count greater than 350 who received MC either immediately or after 2 years. 211 women were in relationships with men in the MC arm and 171 women were in relationships with men in the control group. During 2 years of follow-up, no association with incidence of HPV was detected (IRR = 1.05, 95% CI 0.77-1.43) [[17](#_ENREF_17)]

A large RCT in Uganda enrolled 5,534 HIV-negative uncircumcised men and randomized them to received MC immediately or in 2 years (controls). Their primary outcomes were new HPV, HSV-2 and syphilis infections. Men were followed for two years. The study found that male circumcision was associated with reduced risk of HPV (RR = 0.65, 95% CI 0.46 to 0.90) and HSV-2 (HR = 0.72, 95% CI 0.56 to 0.92) but not syphilis (HR = 1.10, 95% CI 0.75 to 1.65) [[16](#_ENREF_16)] .

### **Do HIV+ men commonly initiate sexual activity before wound healing?**

There is a lack of data on how frequently HIV+ men resume sexually activity before wound-healing. Two studies have some data to address this question and they found that 1.1% and 15.7% of men initiated sexually activity before wound-healing [[10](#_ENREF_10)] [[18](#_ENREF_18)]. Because of the methodologies used, the 1.1% estimate should be viewed as an under-estimate and the 15.7% should be viewed as an over-estimate. Both studies have significant limitations described below. An additional study, also in Rakai, did find that female partners of HIV+ men who resumed sex prior to wound healing had a higher rate of HIV acquisition with 5 of 18 women seroconverting (IRR = 3.50, 95% CI, 1.14-10.76) [[8](#_ENREF_8)].

Details:

A study in Uganda found that 15.7% (66/420) of HIV+ men self-reported initiation of sexually activity before wound-healing was certified as healed. This rate was higher among married men (27.8%). But follow-up, and thus certification of wound-healing, was only done at 24-48 hours, 5-9 days and 4-6 weeks, so this is an over-estimation [[10](#_ENREF_10)].A second study in the same site in Uganda found that 5.5% (10/183) HIV+ men had resumed sexual activity before their week 4 follow-up visit but had not completed would healing during that visit [[18](#_ENREF_18)] .A third study from the site enrolled only HIV+ men age 15-49 who were not stage 3 or 4 and did not have a CD4 count <350[[8](#_ENREF_8)]

### **Time until wound healing**

Three studies looked at time until wound healing comparing HIV+ and HIV- men. Two out of three studies examining men with CD4 counts < 350 found that a smaller proportion of HIV+ men with CD4 count less than 350 had healed by week 4 compared to HIV- men but did not find significant overall differences in healing time between HIV-positive and HIV-negative men . One out of three studies examining only men with CD4 counts >350 found that HIV+ men with CD4 count greater than 350 had slower healing time, and in that study they used the sleeve method. Because the study that found an association between HIV+ men and healing time had the largest sample size of the three studies, and all three studies used different MC methods, it is difficult to make any conclusions.

Details:

A study in Kisumu, Kenya using the forceps-guided method compared the time until healing among men age 18-35 [[19](#_ENREF_19)]. HIV-positive men with signs of Stage 3 HIV or greater were excluded. Men returned weekly to assess if the wound had healed. Comparing 108 HIV+ men to 108 HIV- men, no statistically significant difference in mean time to complete healing was found (33 days for HIV+ vs. 31 days for HIV-). However at week four, 70.4% of HIV- compared to 59.3% of HIV+ men were healed (p=0.09). Among ARV-naïve men, those with CD4 <350 had a mean time to complete healing of 37.1 days compared to those with a CD4 count greater than 31.1 days (p= 0.04). One issue the article raises in the discussion section is that HIV- infection in the first three weeks post-MC is associated with delayed healing, and those individuals should be counseled that they may need to remain abstinent beyond the 42-day period [[19](#_ENREF_19)] .

A study in Uganda using the dorsal slit method compared time until healing among men age 12 and older. 262 HIV- and 177 HIV+ men were enrolled, with participants followed weekly starting at week 3 post-operation. At 4 weeks, 85.9% of HIV- and 77.4% of HIV+ infected men with a CD4 count > 350 and 87.1% of HIV-infected men with a CD4 count less than 350 were healed. The difference between HIV- and HIV+ men with a CD4 count ≥350 was borderline statistically significant (p=0.054). All men in this study had healed by 5 weeks post-surgery [[18](#_ENREF_18)]

A previous study, also in Rakai, Uganda, using the sleeve method enrolled 2,326 HIV- and 420 HIV+ men. They found that at 30 days post-operation, complete wound healing was certified in 73% of HIV+ men and 83.2% of HIV- men (p<0.001), suggesting that HIV- men heal faster. The HIV+ population in this study was restricted to individuals with CD4 count ≥350. 90% of wounds had healed by 6 weeks post-operation [[12](#_ENREF_12)]

In summary, wound healing for those who are HIV+ including those with CD4 <350 does not seem to be very different from those who are HIV-negative.

### **Risks for their partners**

The main risk for partners occurs during the first 6 weeks after MC. One study showed that viral shedding increased during that time, although the increase had subsided in almost all men by six weeks after MC [[20](#_ENREF_20)] . One RCT which attempted to assess whether circumcision in HIV-infected men would reduce transmission of the virus to female sexual partners was stopped early because of futility- 18% of women in the intervention arm and 12% of women in the control arm acquired HIV during follow-up, giving an adjusted HR was 1.49 (0.62-3.57) [[8](#_ENREF_8)]. A meta-analysis of six longitudinal cohort studies also found little evidence that male circumcision directly reduces risk of transmission to women (RR = 0.80, 95% CI 0.53-1.36) [[21](#_ENREF_21)] . With more follow-up time, it is possible that circumcision of HIV+ men is protective for the partner, but there is currently no direct evidence to support this theory.

Details:

A study in Kenya examined penile viral shedding among 20 HIV+, ART-naïve men. Viral shedding generally peaked at week 1, declined steeply in weeks 3-5, and returned to pre-MC levels in week 6. In 28/29 men, no viral shedding was detected after certification of wound healing, so the increased viral shedding was likely due to the local breach in epithelial integrity[[20](#_ENREF_20)].

A RCT of male circumcision in Uganda found no evidence of reduced HIV transmission to female partners. In the study, 922 HIV+ men age 15-49 who were not stage 3 or 4 and did not have a CD4 count <350 men who had uninfected partners were assigned to either immediate or delayed circumcision. Overall, 18% of women in the intervention group (partners in the immediate circumcision group) acquired HIV during follow-up, compared with 12% of women assigned to the control group (partners in the delayed circumcision group). There was no difference in HIV incidence between the circumcised and control groups when the couples waited to resume sex until the wound had healed, which in 93% of male subjects was within 6 weeks of circumcision[[8](#_ENREF_8)].

### **Summary of Studies**

These studies indicate that HIV+ men can undergo surgical circumcision with minimal risk. While there are limited data on those with advanced disease or very reduced CD4 the overall findings are reassuring. Given that PrePex in HIV negative men demonstrates excellent comparability to surgical VMMC, a strong argument can be made to test the safety for PrePex among HIV+ men who are above or below CD4 of 350.

**References:**

1. Njeuhmeli, E., et al., *Voluntary medical male circumcision: modeling the impact and cost of expanding male circumcision for HIV prevention in eastern and southern Africa.* PLoS medicine, 2011. **8**(11): p. e1001132.

2. Tshimanga M, e.a., *A Randomized Controlled Trial Comparing the PrePex™ Device to Forceps Guided Surgical Circumcision for Rapid Scale-Up of Male Circumcision in Zimbabwe. Phase II: Randomized Trial Report*. 2013, Ministry of Health and Child Welfare: Harare, Zimbabwe.

3. Mbonu, N.C., B. van den Borne, and N.K. De Vries, *Stigma of people with HIV/AIDS in Sub-Saharan Africa: a literature review.* Journal of tropical medicine, 2009. **2009**.

4. Glick, P., *Scaling up HIV voluntary counseling and testing in Africa: what can evaluation studies tell us about potential prevention impacts?* Eval Rev, 2005. **29**(4): p. 331-57.

5. Bwambale, F.M., et al., *Voluntary HIV counselling and testing among men in rural western Uganda: implications for HIV prevention.* BMC Public Health, 2008. **8**(1): p. 263.

6. Asante, A.D., *Scaling up HIV prevention: why routine or mandatory testing is not feasible for sub-Saharan Africa.* Bulletin of the World Health Organization, 2007. **85**(8): p. 644-646.

7. Weiser, S.D., et al., *Routine HIV testing in Botswana: a population-based study on attitudes, practices, and human rights concerns.* PLoS medicine, 2006. **3**(7): p. e261.

8. Wawer, M.J., et al., *Circumcision in HIV-infected men and its effect on HIV transmission to female partners in Rakai, Uganda: a randomised controlled trial.* Lancet, 2009. **374**(9685): p. 229-37.

9. Baeten, J.M., et al., *Male circumcision and risk of male-to-female HIV-1 transmission: a multinational prospective study in African HIV-1 serodiscordant couples.* AIDS (London, England), 2010. **24**(5): p. 737.

10. Kigozi, G., et al., *The safety of adult male circumcision in HIV-infected and uninfected men in Rakai, Uganda.* PLoS Med, 2008. **5**(6): p. e116.

11. Herman-Roloff, A., R.C. Bailey, and K. Agot, *Factors associated with the safety of voluntary medical male circumcision in Nyanza province, Kenya.* Bull World Health Organ, 2012. **90**(10): p. 773-81.

12. Kigozi, G., et al., *Safety of Medical Male Circumcision in Human Immunodeficiency Virus–Infected Men in Rakai, Uganda.* Urology, 2014. **83**(2): p. 294-297.

13. Larke, N., et al., *Male circumcision and human papillomavirus infection in men: a systematic review and meta-analysis.* J Infect Dis, 2011. **204**(9): p. 1375-90.

14. Serwadda, D., et al., *Circumcision of HIV-infected men: effects on high-risk human papillomavirus infections in a randomized trial in Rakai, Uganda.* J Infect Dis, 2010. **201**(10): p. 1463-9.

15. Auvert, B., et al., *Effect of male circumcision on the prevalence of high-risk human papillomavirus in young men: results of a randomized controlled trial conducted in Orange Farm, South Africa.* J Infect Dis, 2009. **199**(1): p. 14-9.

16. Tobian, A.A., et al., *Circumcision of HIV-infected men and transmission of human papillomavirus to female partners: analyses of data from a randomised trial in Rakai, Uganda.* Lancet Infect Dis, 2011. **11**(8): p. 604-12.

17. Tobian, A.A., et al., *Male circumcision for the prevention of HSV-2 and HPV infections and syphilis.* N Engl J Med, 2009. **360**(13): p. 1298-309.

18. Kigozi, G., et al., *Male circumcision wound healing in human immunodeficiency virus (HIV)‐negative and HIV‐positive men in Rakai, Uganda.* BJU international, 2014. **113**(1): p. 127-132.

19. Rogers, J.H., et al., *Time to complete wound healing in HIV-positive and HIV-negative men following medical male circumcision in Kisumu, Kenya: a prospective cohort study.* PLoS One, 2013. **8**(4): p. e61725.

20. Odoyo-June, E., et al., *Changes in plasma viral load and penile viral shedding after circumcision among HIV-positive men in Kisumu, Kenya.* J Acquir Immune Defic Syndr, 2013.

21. Weiss, H.A., C.A. Hankins, and K. Dickson, *Male circumcision and risk of HIV infection in women: a systematic review and meta-analysis.* Lancet Infect Dis, 2009. **9**(11): p. 669-77.

22. Bailey, R.C., et al., *Male circumcision for HIV prevention in young men in Kisumu, Kenya: a randomised controlled trial.* Lancet, 2007. **369**(9562): p. 643-56.

23. Gray, R.H., et al., *Male circumcision for HIV prevention in men in Rakai, Uganda: a randomised trial.* The Lancet, 2007. **369**(9562): p. 657-666.

24. Auvert, B., et al., *Estimating the resources needed and savings anticipated from roll-out of adult male circumcision in Sub-Saharan Africa.* PloS one, 2008. **3**(8): p. e2679.

**B. RESEARCH PROCEDURES INVOLVED.**

1. Provide a complete description of: a. the study design, and b. sequence and timing of all study procedures that will be performed, e.g., volume of blood, size of biopsy, drug administration, questionnaire, name of psychological test. Provide this information for each phase of the study (pilot, screening, intervention and follow-up). **Use lay language**. Attach study flow sheet, if available.

- - 1. **Study Design**

This is a one arm, open label, prospective cohort study to assess the safety and acceptability of PrePex™ device circumcision when performed by trained PrePex™ MC Providers in a HIV infected male population. The study will involve 400 HIV+ men. The estimated study timeline of 18 months is:

| **Month** | **1** | **2** | **3** | **4** | **5** | **6** | **7** | **8** | **9** | **10** | **11** | **12** | **13** | **14** | **15** | **16** | **17** | **18** |
| --- | --- | --- | --- | --- | --- | --- | --- | --- | --- | --- | --- | --- | --- | --- | --- | --- | --- | --- |
| **Study Phase** | **Start-up** | | | **Recruitment** | | | | | | **Patient Follow-Up** | | | **Analysis and Report Writing** | | | | | |

The study will have two study components which will be concurrently implemented in an estimated period of 12-18 months. Clinicians and staff involved in the study will follow the standard operating procedures of the study (SOP): Appendix 3.

1. Clinical Component

PrePex™ devices will be applied and removed by trained operators (nurses/doctors) and participants will be followed up over multiple review visits (Procedures and Schedule) to collect data on the clinical adverse events, pain, and time to complete healing. We will compare the rates of AEs among HIV+ men with PrePex MC to the lower historical proportion of men who experience an AE with surgical MC of 2.0% as seen in Kenya [[22](#_ENREF_22)] and not to the higher rates of AEs of around 3% as seen in both Uganda [[23](#_ENREF_23)] and South Africa [[24](#_ENREF_24)]. Additionally, we will qualitatively compare this data to those collected in Zimbabwe from two previous studies and routine surveillance in Zimbabwe: 1. a comparison group of HIV-uninfected men who were recently circumcised by the same MC providers using the PrePex device; 2. a comparison group of HIV-uninfected men who recently underwent routine VMMC by MC providers using the forceps-guided method.

1. Psychosocial Component

Individual interviews will be conducted by trained psychosocial interviewers to collect data from HIV-infected participants on satisfaction with the PrePex™ MC procedure, attitudes towards MC, compliance with instructions to refrain from sexual intercourse for 7 weeks post placement [(Day 49 or 6 weeks post removal) or until a clinician tells them that they are fully healed – whichever is later], barriers and motivators to uptake of VMMC through PrePex™, opinions around convenience of the device, tolerance of potential odour and whether participants would recommend PrePex™ MC to others. The interviews with same participants will be held at three points in time: prior to the procedure, two weeks post-procedure, and 90 days post-procedure (Appendices 6-8). In addition all health care providers at facilities where PrePex™ circumcision is offered will be interviewed on the acceptability of the PrePex™ procedure for HIV-infected clients. Interviews with providers (Appendix 9) will take place at one time point approximately 4 weeks post study initiation.

**Study Setting**

The PrePex device procedure will be performed by the same set of nurses/doctors at two sites. Follow-up visits may also occur in participants’ homes if the patient fails to return for the scheduled visit on Day 7, removal visit.

- Chitungwiza Hospital: This site will be set up as a new training centre to increase country capacity to train trainers of PrePex™ operators and assistants.
- Zengesa clinic in Chitungwiza: This is an established site that provides surgical VMMC
- Recruitment only sites: Opportunistic infection clinics at Beatrice and Wilkins hospitals. Recruiting scripts will be followed (Appendix 10)

**Study Duration:**

The study may last up to 18 months. Participants will be followed for 8 weeks to allow for an assessment of time until complete wound healing. or until declared fully healed by a clinician, whichever is later. Men selected for interviews will also have a visit 90 days after the MC procedure.

- - 1. **Sequence and Timing of Study Procedures:**

All study procedures will be implemented in 12-18 months as shown in the schedule (Table 1) and will include weekly review visits to assess AEs and time to complete healing.

**Procedures and Schedule**

The study schedule of events is summarized in Table 1. Each participant will undergo a series of study visits including the screening visit, device application visit, device and foreskin removal visit and additional follow-up visits up to 8 weeks. Below is a detailed description of the visits.

**Screening Visit**

- Administer informed consent (Appendix 1)
- Rapid HIV test: to assess HIV status as an eligibility criterion
- Case Report Forms (CRFs) (Appendix 4) completed by clinician which includes:
  - Clinical exam
  - Medical History
  - Current medications
  - Finger prick for blood drop collection for CD4 count by PIMA Point of Care CD4 machine (if HIV positive)

**Device Placement: Day 0**

- Client Interviews
- Case Report Forms (CRFs) (Appendix 5) completed by clinician which includes:
  - PrePex™ sizing
  - Procedural notes
  - Occurrence of any adverse events
  - Evaluation of initial patient pain level after device application
  - Hygiene instructions for penis care during device wearing period (Appendix 15)

The procedure will be carried out in a clean non-sterile environment. The subject will undergo sizing of the penis sulcus with the PrePex™ Sizing Plate. The penis will be cleaned by red Providine-Iodine solution or other disinfection solution (Chlorahexidine,etc.) with the foreskin stretched backwards to expose the inner skin for the disinfection solution. Then the foreskin will be dried with a dry gauze. The Operator will then mark the circumcision line with a clean skin marker and then the Assistant will open the corresponding sized package of the PrePex™ device, and will load the Elastic Ring on to the Placement Ring. Then, Lidocane 5% dermal cream will be applied on the inner foreskin. The Placement Ring with the Elastic Ring will be placed on the penis shaft and the foreskin tip will be stretched open to the sides by the operator hands, the operator may select to use dry gauze to affirm the grip. Then, the Inner Ring will be applied through the stretched foreskin and over the glans penis (hereinafter, "glans") so that it will be placed fixed over the sulcus behind the glans (the base of the glans). At this point, the foreskin around the inner ring is held tight by the Assistant, and the Placement Ring and Elastic-Ring are approximated to the Inner Ring by the Operator. The foreskin is then adjusted to fit the circumcision marking line to the Elastic-Ring. Then, the Elastic-Ring is released from the Placement Ring on to the Inner Ring groove, initiating homeostasis. The procedure is then completed. Medication based on 400mg Ibuprofen is given to the subject to use at home if needed (or Paracetamol [Tylenol] if Ibuprofen is not appropriate for the patient). The subject will then take part in a formal discharge session in a class room, where he will be instructed to return for a follow up visit and in any non-sustained situation and to take painkillers in case of pain. If an infection is identified at any of the follow-up visits, patients may be treated with the antibiotics Cloxacillin or Ciprofloxacin. All patients will be given oral instructions for hygiene in Prepex post insertion care, adapting the device instructions for the local context (Appendix 15).

**Day 7** : **Device removal**

- The PrePex™ device is removed
- Dead skin is removed using sterile scissors
- Sterile gauze and adhesive tape will be applied to the wound
- Case Report Forms (CRFs) (Appendix 5) completed by clinician which includes:
  - Clinical exam
  - Evaluation of patient pain level during and after device removal
  - Occurrence of any adverse events
  - Current Medications
  - Photograph of the penis taken if consent granted
  - Reminders to abstain from sexual intercourse and masturbation
- Preparation and procedure time may be measured by stopwatch

**Day 9**

- The dressings on the wound are removed
- Case Report Forms (CRFs) (Appendix 5) completed by clinician which includes:
  - Clinical exam
  - Evaluation of patient pain level
  - Occurrence of any adverse events
  - Current Medications
  - Photograph of the penis taken if consent granted
  - Reminders to abstain from sexual intercourse

**Day 14**

- Client Interviews
- Case Report Forms (CRFs) (Appendix 5) completed by clinician which includes:
  - Clinical exam
  - Evaluation of patient pain level
  - Occurrence of any adverse events
  - Current Medications
  - Photograph of the penis taken if consent granted
  - Reminders to abstain from sexual intercourse
  - Assessment of wound healing

**Day 21, Day 28, Day 35, Day 42, Day 49, Day 56, Day 63, Day 90**

Patients will be followed in weekly visits until Day49 or until determined fully healed by a clinician (whichever is later). Visits will also occur on Day 90 for those men participating in the psychosocial questionnaire. In each follow-up visit other than Day 90, the following will occur:

- Case Report Forms (CRFs) (Appendix 5) completed by clinician which includes:
  - Clinical exam
  - Evaluation of patient pain level
  - Occurrence of any adverse events
  - Current Medications
  - Photograph of the penis taken if consent granted
  - Reminders to abstain from sexual intercourse until fully healed
  - Assessment of wound healing

**Final Visit : Day 90**

- Client Interviews for those selected for interview
- Final Medical Assessment
- Case Report Forms (CRFs) completed by clinician which includes:
  - Final Medical Assessment
  - Patient satisfaction assessed

**Patient Follow-up**

If a participant fails to attend the scheduled weekly visit, he will be contacted by phone and/or text and will be asked to attend. The provider will make at least three attempts to contact the participant if he does not return to the study site for any study visit. Starting one day after a missed Day 7 (removal) visit, study participants will be called and texted. Study staff will attempt a home visit starting at two days post a missed visit for Day 7, removal visit. For other missed visits, only phone calls/texts will be utilized. Home visits will be used only for a missed removal visit. Participants lost to follow-up will not be replaced. All attempts to contact the client will be recorded on a contact log in the client’s file.

Patients may make unscheduled visits if they are concerned about adverse events, require review, or come in before or after a scheduled appointment. Unscheduled visit forms and adverse event review forms accommodate these possibilities.

If complications or delayed healing require longer follow-up, men will be followed by study staff as long as necessary or until study close-out. If clinical care is required after study close-out, care will be provided at the study site as part of regular clinical service. If necessary, men may be referred to a hospital near the study site for additional care.

**Patient interviews:**

For the interviews, up to 103 men who get circumcised and consent will be randomly selected to have a private *face-to-face interview* at three points in time: prior to their procedure, 14 days and 90 days after procedure. Interviewer administered questionnaires will be used to collect required data.

**Healthcare worker interviews:**

Among healthcare workers, all healthcare providers involved in this PrePex study will given the opportunity to complete interviews on their opinions and views on the PrePex™ device. All healthcare workers will be consented (Appendix 2); participation is voluntary. These interviews will enable us to evaluate the acceptability of the PrePex™ procedure from service providers. Interviews will take place approximately 4 weeks after study implementation begins.

**Table 1: Study timeline**

| **Table 1: Protocol Description and Timing of Assessments** | | | | | | | | |
| --- | --- | --- | --- | --- | --- | --- | --- | --- |
|  | **Screening and Recruitment Visit** | **Application Visit** | **Removal Visit** | **Physical Examination of the genital Visit** | **Physical Examination of the genital Visit** | **Physical Examination of the genital Visit** | **Physical Examination of the genital Visit** | **Physical Examination of the genital Visit** |
| **Visit #** | **1** | **2** | **4** | **5** | **6** | **7** | **8** | **9, 10, 11, 12, 13** |
| **Day (D)/week (w)** | **–7 to D0** | **D0** | **D7** | **D9** | **D14** | **D21** | **D28** | **D35, 42, 49, 56, 63** |
| Informed consent | X |  |  |  |  |  |  |  |
| Screen with Inclusion/exclusion criteria | X |  |  |  |  |  |  |  |
| Participant specimen collection for CD4 count | X |  |  |  |  |  |  |  |
| Medical history & Demography | X |  |  |  |  |  |  |  |
| Current medication including pain killers | X | X | X | X | X | X | X | X |
| Psychosocial interview |  | X |  |  | X |  |  | X (D90) |
| Genital examination and potential pictures | X | X | X | X | X | X | X | X |
| Video procedure |  | X | X |  |  |  |  |  |
| Device application |  | X |  |  |  |  |  |  |
| Foreskin removal |  |  | X |  |  |  |  |  |
| Device removal |  |  | X |  |  |  |  |  |
| Subject's subjective pain, tingling and discomfort evaluation (VAS score) |  | X | X | X | X | X | X | X |
| Evaluate side effects and AE |  | X | X | X | X | X | X | X |
| Statistical analysis of data |  |  |  |  |  |  |  | Interim safety report |

2. Would subjects undergo these or similar procedures (medical, psychological, educational, etc.) if they were not taking part in this research?  No  Yes If “Yes,” describe how the study procedures differ from what subjects would otherwise undergo.

HIV+ men are not excluded from VMMC according to the Zimbabwe MOHCC, but the program is intended as an HIV prevention program and focuses on HIV-negative men. The PrePex™ device was approved by the Zimbabwe MOHCC for use among HIV-negative men ages 18 and older, and routine implementation began in early 2014. This protocol follows the standard protocols for this activity, extending the target group to include HIV-positive men. Men participating in this study are currently eligible for forceps-guided MC. As with all MOHCC VMMC program services, participants in the study will also receive VMMC at no cost.

3. Check all of the boxes below that apply to your research:

**Drug administration**

Administration of a drug (either FDA-approved or investigational) for research purposes to a subject-patient during general or regional anesthesia.

Administration of a drug (either FDA-approved or investigational) for research purposes to a subject-patient during the 1.5 hours preceding general or regional anesthesia.

**Blood lines**

Inserting an intravenous (central or peripheral) or intra-arterial line for research purposes in a subject-patient **during** general or regional anesthesia.

**Sample collection**

Obtaining samples of blood, urine, or cerebrospinal fluid for research purposes while a subject-patient is under general or regional anesthesia.

Obtaining a research sample from tissue or organs that would not otherwise be removed during surgery, while the subject-patient is under general or regional anesthesia.

**Radio-isotopes**

Administration of a radio-isotope for research purposes during the 3 hours prior to anesthesia or while a subject-patient is under general or regional anesthesia.

If you checked this box, you are responsible for informing **in advance** all appropriate clinical personnel (e.g., nurses, technicians, anesthesiologists, surgeons) about the administration and use of the radio-isotope, to ensure that any personal safety issues (e.g., pregnancy) can be appropriately addressed. This is a condition of IRB approval.

**Experimental devices**

Implantation of an experimental device while a subject-patient is under general or regional anesthesia.

**Other experimental manipulations or procedures**

Other manipulations or procedures performed solely for research purposes while a subject-patient is under general or regional anesthesia (e.g., experimental liver dialysis, experimental brain stimulation)

**None of the above**

None of the above apply to my research

4. If you checked any box in question #3 except “none of the above”, answer the following questions:

a. Provide the name and institutional affiliation of the physician anesthesiologist who is a member of your research team or who will serve as a safety consultant about the interactions between your research procedures and the general or regional anesthesia of the subject-patients. If your procedures will be performed at a UW Medicine facility or affiliate, the anesthesiologist must be a UW faculty member.

Not applicable

b. If you have not yet consulted with an appropriately qualified person about this issue, describe in detail your plans to do so. The IRB will not approve your application without this consultation. If UW Department of Anesthesiology approval has been obtained, please provide the Department’s letter of support.

Not applicable

**C. DECEPTION**: If any deception or withholding of complete information is required for this activity, explain why this is necessary and attach a protocol explaining if, how, when, and by whom subjects will be debriefed.

There is no deception or withholding of completed information required of this activity.

**D. SUBJECTS**

The IRB reviews the number of subjects you plan to study in the context of risks and benefits. If your research is approved for a specific number of subjects, the data from any “extra” subjects cannot be described as having been obtained with IRB approval.

See the HSD website for the definition of “human subject” <http://www.washington.edu/research/hsd/docs/1253>. Before answering the questions below, be sure that you are familiar with the definition.

1. **Subject groups/categories and numbers.** Complete this table by listing:

- Your groups or categories of subjects. “Group” should be defined as appropriate for your research.
  - “Units” within a group. For most research, a group will consist of individuals, such as children aged 8-12, or individuals with high blood pressure. However, this will not be true for all research. Examples of groups with “units” that are not individuals:
    - Dyads such as Alzheimer’s-patient-and-caregiver, with one group of the dyads assigned to one intervention (e.g., behavioral modification) and another group of the dyads assigned to a comparison intervention(e.g., drug treatment).
    - Families. For example, a study of mental health interventions for homeless families might have one group of 30 families assigned to one intervention and another group of 30 families assigned to a different intervention.
    - Other. For example, the “units” in autism research might be an autistic individual and all his/her living blood relatives. The units in an academic excellence study might be a student-parents-teacher unit.
  - Types of groups. There are many ways in which subjects might be grouped. Examples:
    - By intervention. Example: research comparing two different drugs for high blood pressure.
    - By subject population. Example: research comparing the incidence of domestic violence in families living in urban settings versus families living in rural settings.
    - If you have only one group, fill in only one line in the table. Add more lines if needed.
- The age range of each group.
- The upper limit/number of **completed** subjects you need for each group. ***Completed*** *means that all research procedures involving the subjects or the obtaining of specimens/records/data have been completed as far as is possible for each subject, including any follow-up (such as follow-up access to medical records.) In some cases, such as an online survey, it is not possible to predict the number of subjects who will complete the research. If you cannot predict or describe the maximum number of subjects you need in each group, check the appropriate box and provide your rationale in the space provided below the table.*

| **Group name/description for routine program activities** | **Age range of subjects** | **Maximum desired number of individuals (or other group unit, such as families) who will complete the research.*** | **Cannot provide a number.**** |
| --- | --- | --- | --- |
| HIV-positive VMMC clients (males) | 18+ years | 400 |  |
| Healthcare providers | 18+ years | Up to 20 | ****** |
|  |  |  | ****** |
|  |  |  | ****** |

***This is the number of subjects (individuals, dyads, families, etc., as appropriate) in each group that will be considered for approval by the IRB.**

****If you cannot predict or describe the maximum number of subjects you need in each group:**

Provide your rationale and description of research scope here. *Include any information or estimates you might have about the number of subjects, so that the IRB has a sense of the scope of your research. For example, your research might be a small pilot study of all patients presenting with a rare disease at UW Medicine in the next year. Or, it might involve a survey posted on Craig’s List for two weeks that could result in thousands of responses.*

| **NOTE: In your annual Status Report, you will be asked to complete the table below with your subject numbers. While developing your research protocol, please plan ahead so that you will have an accurate record of the subject numbers above.**  **This is for illustration only. Do not complete this table.**   \|  \| # Completions \| \| \| \| # Ongoing (subjects still involved) \| # Withdrawals, drops, lost \| \| \| \| --- \| --- \| --- \| --- \| --- \| --- \| --- \| --- \| --- \| \| Group Name / Description \| Total approved by IRB \| A At time of last Status Report \| B Since last Status Report \| A + B Total to date \| C At time of last Status Report \| D Since last Status Report \| C + D Total to date \| \|  \|  \|  \|  \|  \|  \|  \|  \|  \| \| DO NOT COMPLETE \|  \|  \|  \|  \|  \|  \|  \|  \| \|  \|  \|  \|  \|  \|  \|  \|  \|  \| \|  \|  \|  \|  \|  \|  \|  \|  \|  \| |
| --- | --- | --- | --- | --- | --- | --- | --- | --- | --- | --- | --- | --- | --- | --- | --- | --- | --- | --- | --- | --- | --- | --- | --- | --- | --- | --- | --- | --- | --- | --- | --- | --- | --- | --- | --- | --- | --- | --- | --- | --- | --- | --- | --- | --- | --- | --- | --- | --- | --- | --- | --- | --- | --- |

2. Explain how you will achieve equitable subject representation in the following categories. If not applicable, justify exclusions.

The Zimbabwe MOHCW has determined the eligibility criteria for VMMC. I-TECH operates as an implementer of their national program. Those criteria are:

a. Age (minors, elderly): N/A: The VMMC program using PrePex™ is open to all men ages 18+, with a specific target for HIV- sexually active men, ages 18-29. Men in this study will be recruited if they are HIV-positive and meet all other study eligibility requirements.

b. Gender: The VMMC procedure is only available for men

c. Ethnic and racial minority populations: We anticipate the ethnic and racial distribution of the study population will reflect the ethnic and racial distribution of men willing to participate in the VMMC program in the areas in Zimbabwe we are working in.

3. What characteristics (inclusion criteria) must subjects have to be in this study? (Answer for each subject group, if different.)

Inclusion Criteria:

- Agrees to take an HIV test
- HIV sero-positive
- WHO HIV clinical stage 1 or 2
- Age 18 years and above
- Agrees to be circumcised by PrePex™
- Able to understand the study procedures and requirements
- Completes VMMC counseling
- Agrees to return to the health care facility for follow-up visits (or as instructed) after his circumcision until complete healing 7 weeks from device application (Day 49 visit) or until cleared by a clinician, whichever is later
- Able to comprehend and freely give informed consent for participation in this study

4. What characteristics (exclusion criteria) would exclude subjects who are otherwise eligible from this study? (Answer for each subject group, if different.)

Exclusion Criteria:

- Known bleeding/coagulation abnormality (excessive bleeding from nosebleeds, pulled tooth, or gums)
- Uncontrolled diabetes (frequent treatment in a clinic or recent hospitalization for diabetes, frequent infections, hypertension, or kidney disease),
- Active genital infection, anatomic abnormality or any other condition, which in the opinion of the investigator prevents the subject from undergoing circumcision using PrePex.
- HIV sero-negative
- HIV status unknown
- WHO HIV stage 3 and above
- Latex allergy
- Does not agree to PrePex

5. Describe the subject recruitment strategies you will use for each group of subjects. (Attach advertisements, flyers, contact letters, telephone contact protocols, Health Sciences recruitment web site template, etc.)

Study staff may identify potential participants from any of the following sites: OI and VCT centres, Zengeza clinic, Chitungwiza hospital, and OI clinics at Beatrice and Wilkins hospitals. After learning about the study, those who agree to be circumcised using PrePex will be requested to visit Zengeza clinic for further screening by the study coordinator using eligibility criteria before actual recruitment into the study. Services at the study site, Zengeza clinic, will be offered every day from 8:00 am to 4:00 pm except on Sunday.

6. Explain who will approach subjects to take part in the study and how this will be done to protect subjects’ privacy. (Attach letters of cooperation from agencies, institutions or others involved in subject recruitment.)

Participants will be recruited from any of the following sites: Voluntary testing and counseling (VCT) centers at Zengeza clinic and Chitungwiza hospital and opportunistic infection clinics (for HIV-positive patients) at Beatrice and Wilkins hospitals.

Trained counsellors at selected OI clinics or VCT centres will help identify potential participants by asking all men presenting to these sites if they would be willing to be contacted by a study coordinator to set an appointment for PrePex MC screening. Men seeking care at OI clinic (existing HIV-positives) and men who test positive at these sites (new HIV-positives) after undergoing HTC according to Ministry of Health (MOHCC) guidelines (including pre- and post-test counseling) may be recruited. For men who are not confirmed in HIV-related care or do not have a confirmed, recent (within 4 weeks) HIV+ test result from a facility or testing station, they will undergo an HIV test using the Ministry of Health and Child Care (MoHCC) guidelines that include pre- and post-test counseling to confirm their HIV status as an eligibility criterion. Consent for HIV testing will be done according to MoHCC guidelines. Men aged 18 years or older, who are eligible for circumcision using the PrePex^TM^ device, and meet the inclusion criteria, will be included in the study. All eligible men will be invited to participate until the required sample size is achieved. Participants will be asked to provide their phone numbers and their written consent to be contacted over the phone and to be followed-up by home visit.

7. Explain what steps you will take during the recruitment process to minimize potential coercion or the appearance of coercion.

ZiCHIRe will train community mobilizers and health care workers on the ethics and principles of preventing coercion and maintaining confidentiality. Potential HIV+ VMMC clients will be informed that the procedure is voluntary, and they will be required to sign a consent/assent form to participate. Potential clients will also be informed they can withdraw from participation at any time for any reason, without penalty or prejudice, and will be informed that declining to participate in this study will not jeopardize their access to routine health are or VMMC services in Zimbabwe.

8. Will you give subjects gifts, payments, services without charge, or extra course credit?  No  Yes If yes, explain:

Participants will be compensated for their time and effort in this study. They will receive approximately US$6 per visit to take care of transport reimbursement, refreshment and client's time. Healthcare workers will receive $10 at time of interview. The reimbursement amounts are a requirement by Medical Research Council of Zimbabwe and were provided for participants in previous PrePex™ trials, including the ongoing trial for adolescents led by the same Zimbabwe PI.

9. Will any of the subjects or their third-party payers be charged for any study procedures?  No  Yes If yes, explain:

10. **Locations and research sites.** Provide the following information in list or table format for all locations at which any research procedures will occur. Be sure to consider: screening, recruiting, consenting, observation, intervention, data collection, data analysis, specimen analysis, and location of any consultants and collaborators. Recruiting script (Appendix 10) and flyer (Appendix 16) are attached.

- Geographical location and/or address
- Name of organization, agency, group, site, institution
- What procedures will occur at each location (how the location is involved in the research)
- Whether subject contact or interaction will occur at each site
- Whether consenting of subjects will occur at each site
- Whether each site, or individuals at the site, will obtain, use, or have access to coded or individually identifiable private information about subjects for research purposes

| **Site** | **Recruitment** | **Screening/Consent** | **PrePex Procedure** | **Follow-up/AE Response** | **Data Storage** | **Data analysis** |
| --- | --- | --- | --- | --- | --- | --- |
| Chitungwiza Hospital | X | X | X | X | X |  |
| Zengeza clinic in Chitungwiza | X | X | X | X | X |  |
| OI clinic at Beatrice Hospital | X |  |  |  |  |  |
| OI clinic at Wilkins Hospital | X |  |  |  |  |  |
| Zichire office, Harare, Zimbabwe |  |  |  |  | X | X |
| I-TECH/Seattle |  |  |  |  | X | X |

The PrePex™ device procedure will be performed by nurses/doctors at Chitungwiza Hospital and/or Zengeza clinic in Chitungwiza, outside of Harare Zimbabwe. Chitungwiza Hospital will be set up as a new training centre to increase the country capacity to train trainers of PrePex™ operators and assistants. Sites will have a PIMA machine so that clients can have their CD4 tested on site with immediate results. All study activities will take place at these sites from consent, procedure, interviews and all follow-up visits.. Sites will house all data and study-related information that includes names. Data management and confidentiality procedures are in place. Recruitment activities will take place at OI and VCT centers near the city of Chitinguwiza, including Zengeza clinic, Chitungwiza hospital and OI clinics at Beatrice and Wilkins hospitals. Analysis will take place at the Zichire office in Harare and in I-TECH/Seattle.

**Data Collection**

Overview table:

| Study Objective | Sample Size Est. | Sampling method | Data collection Methods | Tools |
| --- | --- | --- | --- | --- |
| 1.1.1 (Safety) | 400 | Purposive | Interview | CRF |
| 1.2.1 (Level of care) | 400 | Purposive | Interview  Observation | CRF |
| 1.2.2 (Acceptability) |  |  |  |  |
| Clients | 103 | Random | Interview | QES |
| Providers | All | Purposive | Interview | QES |
| 1.2.3 (Facilitators/Barriers) |  |  |  |  |
| Clients | 103 | Random | Interview | QES |
| Providers | All | Purposive | Interview | QES |
| 1.2.4 (Compliance with instructions) | 103 | Purposive | Interview | QES |
| 1.2.5 (AE monitoring) | 400 | Purposive | Interview  Observation | CRF |

If you are the direct recipient of federal funding for this project and other organizations are “engaged” in the research (see the Worksheet on Engagement), you are responsible for obtaining IRB approval from the other organizations or for requesting that the UW conduct the IRB review on behalf of that organization. See the document [SOP Research Collaborations or Multiple Performance Sites](http://www.washington.edu/research/hsd/docs/1644) and [WORKSHEET IRB Review Options](http://www.washington.edu/research/hsd/docs/1645) for more information.

**E. RISKS AND BENEFITS**

1. Describe nature and degree of risk of possible injury, stress, discomfort, invasion of privacy, and other side effects from all study procedures, drugs and devices (standard and experimental), interviews and questionnaires. Include psycho-social risks as well as physiological risks. Include risks of withholding standard care or procedures if this is the case. Do not reference the consent form.

There is potential increased risk of male to female HIV transmission during the period of wound healing. Participants will be repeatedly advised to abstain from any sexual activity before complete wound healing. Patients will be reminded at each visit that abstinence is the safest way to avoid transmission and support wound healing. However, if the man cannot abstain, condoms should always be used or other forms of sexual intimacy should be practiced until the wound is fully healed.

As a routine medical preventive procedure, MC via PrePex includes standard risks which are minimized and managed via standard care and follow-up; any additional MC-related risk that may be incurred by participants by virtue of their HIV status will be monitored closely and managed as described above. There are other minimal risks of participating in this study such as feeling of discomfort or little pain due to needle prick to collect blood for CD4 counts. Participants will also be informed that they can withdraw from the study at any time with no repercussions.

**Adverse Events**

Although unexpected, potential risks from circumcision include excessive bleeding, hematoma, infection, pain, wound dehiscence, injury to the glans or urethra, excessive or insufficient skin removal, and poor cosmetic results. The use of the PrePex™ device has yielded low rates of AEs in the clinical studies conducted in Rwanda. In the safety study in Rwanda, all 50 procedures were completed successfully: only one mild AE, edema, was reported among the 50 participants and mean pain scores were low. There were no reports of infection, bleeding, hematoma, delayed necrosis or wound dehiscence. There were several cases of mild oozing from the wound and lesser edema cited, without being classified as AEs. In the randomized comparison study in Rwanda, among 144 PrePex™ clients, one moderate and three mild AEs were reported (2.7%) but none were device related, and all resolved with minimal intervention. Similar AE rates are expected in this Zimbabwe study; however, AE and complication rates following PrePex™ circumcision among HIV+ men will be further clarified in this study.

Throughout the studies conducted using the PrePex™ device to date, there have been instances when the device components have become displaced (the inner ring and/or outer plastic band shifts from its original placement position). Sometimes, such complications were clinically managed by repositioning the device components or removing the device entirely. Alternatively, in some cases of displacement, repositioning or removal of the device components was not an option, and a surgical circumcision was required to appropriately manage the situation. The clinical management of displacement (surgical circumcision vs. repositioning vs. removal) depends upon the time post-application that the displacement occurs. Serious displacements requiring surgical correction have been rare overall (<0.5%). Because all cases were recognized and treated promptly and appropriately, all such clients have had a full recovery. Study procedures are in place to detect and manage cases of device displacement. Study participants are also educated about the need to avoid activities that might lead to displacement of the device, the risks of the device becoming displaced and the need to immediately contact the study site in the event of displacement for any reason.

There may be a potential for post-procedure risk compensation in this study (i.e. after circumcision) and men might engage in riskier sexual behaviors. However, there is no evidence to date that risk compensation is a major problem following adult MC in a number of sub-Saharan African countries, and this risk is present following circumcision done by any method.

2. Explain what steps you will take to minimize risks of harm and to protect subjects’ rights and welfare. (If you will include protected groups of subjects (minors, fetuses in utero, prisoners, pregnant women, decisionally impaired or economically or educationally disadvantaged subjects) please identify the group(s) and answer this question for each group. Please also complete the [Supplement: Protected and Vulnerable Populations](http://www.washington.edu/research/hsd/docs/322).)

The study will be conducted by the team comprised of the same cadres of staff that carried out previous PrePex studies in Zimbabwe. It comprises of two PrePex master trainers including one surgeon, one urologist and four trained nurses. They will be given an update on the minimum package of VMMC services which includes: information about the risks and benefits of the MC procedure, counselling about the need to adopt and maintain safer sexual practices, access to HIV testing, condom promotion and provision and the management of sexually transmitted infections (STIs). They will also be reminded about the increased risk of HIV transmission and the need to adopt strict infection control measures.

VMMC, PrePex™ procedure – Management of Adverse Events: The participant will be instructed to return to the study site or the nearest hospital (in the case of dislodgement or severe pain) in case of any unexpected event, for example if there is severe discoloration of the penis, signs of infection (purulence), severe or increasing pain, if any part of the device moves for any reason, if the distal part of the foreskin detaches, or any other significant concern that they may have The participant will be reminded to abstain from sex and to avoid masturbation. Clients will be instructed that if the device becomes displaced (moves) for any reason that he must seek medical care at a hospital immediately, and that if the device components become displaced and the client doesn’t return immediately for care, there is a risk of permanent damage to his penis. In the event of a complication meeting the case definition for a clinical AE associated with the VMMC, the provider will document the complication in the register according to the guidelines. Mild post-operative infections will be managed with wound cleansing and dressing, and antibiotics will be prescribed (see medications list) where clinically indicated. If device components become displaced, a surgical circumcision may be required.

Confidentiality: The following procedural efforts will be made to avoid breaches in confidentiality. All medical and programmatic data collected will be stored in a locked file cabinet, room or on password protected computers, with access provided only to authorized project staff. No one outside the investigators and team listed on the protocol or their designates will have access to the de-identified data. All investigators and analysis team members will have to sign a data use and confidentiality agreement.

3. Is it possible that you will discover a subject’s previously unknown condition (disease, suicidal intentions, genetic predisposition, etc.) as a result of study procedures?  No  Yes If yes, explain how you will handle this situation.

As a result of this study, it is possible that we will discover unknown medical conditions through our medical examination and HIV screening. However, as we are recruiting at HIV clinics, we expect all recruits to be previously diagnosed and informed of their HIV status. If any unknown condition is discovered as a result of this study, the subject will be referred for treatment and care as per Government of Zimbabwe guidelines.

4. Describe the anticipated benefits of this research for individual subjects in each subject group. If none, state “None.”

Participants will directly benefit from the advantages of male circumcision. Information obtained in this study will be used by the MOHCC and its partners to inform the rollout of male circumcision using the PrePex™ device among HIV+ men in Zimbabwe which may lead to additional health benefits for participants and their partners, as well as improving MC coverage and thus HIV prevention among men unwilling to test for HIV who are actually still HIV-negative.

Compensation for travel and time:

5. Describe the anticipated benefits of this research for society, and explain how the benefits outweigh the risks.

The findings of the study will be used primarily to inform the roll-out of VMMC services for HIV positives using the PrePex™ device in Zimbabwe and to provide critical guidance to develop strategies to increase uptake of VMMC devices among HIV positive men. The findings will also be used to make recommendations regarding training of clinicians and messages to be conveyed to HIV positive participants in pre-procedure counseling sessions. Ultimately, these findings will contribute to the body of knowledge required to obtain WHO pre-qualification for the use of this device in HIV positive men in Zimbabwe and across the sub-region.

**F. ADVERSE EVENTS OR EFFECTS**

1. Who will handle adverse events?  Investigator  Referral  Other, explain:

The participant will be instructed to return to the study site or the nearest hospital (in the case of dislodgement or severe pain) in case of any unexpected event, for example if there is severe discoloration of the penis, signs of infection (purulence), severe or increasing pain, if any part of the device moves for any reason, if the distal part of the foreskin detaches, or any other significant concern that they may have. The participant will be reminded to abstain from sex and to avoid masturbation until 7 weeks post placement or until told that they are fully healed by a clinician, whichever is later. Clients will be instructed that if the device becomes displaced (moves) for any reason that he must seek medical care at a hospital immediately, and that if the device components become displaced and the client doesn’t return immediately for care, there is a risk of permanent damage to his penis.

In the event of a complication meeting the case definition for a clinical AE, the provider will document the complication in the register according to the guidelines. Mild post-operative infections will be managed with wound cleansing and dressing, and antibiotics will be prescribed (see medications list) where clinically indicated. If device components become displaced, a surgical circumcision may be required.

If complications or delayed healing require longer follow-up, men will be followed by study staff as long as necessary or until study close-out. If clinical care is required after study close-out, care will be provided at the study site as part of regular clinical service. If necessary, men may be referred to a hospital near the study site for additional care.

2. Are your facilities and equipment adequate to handle possible adverse events? Yes  No, explain:

3. Who will be financially responsible for treatment of any **medical problems or physical side effects** that appear to be caused by the study**,** including any effects of a blood draw (such as a local infection)?

Not applicable; there are no risks of medical problems or physical side effects.

Department of Defense (DoD) or a DoD component (for example, the Navy).

*Select this box if there are possible medical problems or physical side effects* ***and*** *the research is directly funded by the DoD or a DoD component.*

The VA (Veterans Affairs)

*Select this box if there are possible medical problems or physical side effects* ***and*** *the study will be conducted at the VA with VA patients. If the study will be conducted at both the VA and the UW, and it will involve both VA and UW patients, select this box* ***and*** *the UW Compensation Program.*

The study sponsor

*Select this box if there are possible medical problems or physical side effects* ***and*** *there is a contract or other written agreement by which the study sponsor agrees to pay. This option generally applies to UW-conducted research only when the research is an industry-sponsored clinical trial that involves protocols initiated and designed by a company rather than by the UW investigator. It may also apply to federally-funded UW vaccine trials that are eligible for the National Vaccine Compensation Program.*

The UW Human Subjects Compensation Program

*Select this box if there are possible medical problems or physical side effects* ***and*** *(1) the investigator is a UW employee or student; and (2) all funding (if any) for the research is administered through the UW Office of Sponsored Programs or is coming from internal UW funds.*

- ***This is the option that applies to most studies reviewed by the UW IRB.***
- ***This option does not apply to studies conducted by investigators at non-UW institutions (such as Puget Sound Blood Center) even when those investigators have UW faculty appointments, unless the research funding is administered through the UW Office of Sponsored Programs.***

Subject or subject’s insurer

*Select this box only if none of the above options are applicable.*

Describe and explain why this is the most appropriate option:

The Zimbabwe National Health System will absorb the cost of any medical problems or side effects. This is the most appropriate option because the National Health System absorbs the cost of medical problems arising from the MOHCC VMMC program, of which this study is a part.

Other

Describe and explain:

**G. CONFIDENTIALITY OF RESEARCH DATA**

1. Will you record any direct subject identifiers (names, Social Security numbers, patient, hospital, laboratory or claim numbers, addresses, telephone numbers, locator information, etc.)  No  Yes If yes, explain why this is necessary and describe the coding system you will use to protect against disclosure.

Zimbabwe MOHCC standard Monitoring &Evaluation forms contain patient identifiers for record keeping and patient follow-up including location of household and cell numbers. All records used for this intervention that are based on these registers will follow standard operating procedures employed by the Zimbabwe MOHCC including maintaining registers and patient forms in secure facility locations. Specific supplemental forms with personal identifiers, including those used for active follow-up, will be stored in a locked cabinet and electronic files will be password protected. CRFs will only contain participant identification number (PIN#), not name, to help protect confidentiality. National ID or MC ID, routine components of Zimbabwe MOHCC forms, will be used to link patients. Other study-related unique codes will not be created. Password protected computers and locked file cabinets are the standard of study operations.

2. Will you retain a link between study code numbers and direct identifiers after the data collection is complete?  No  Yes If yes, explain why this is necessary and for how long you will keep this link.

3. Describe how you will protect data against disclosure to the public or to other researchers or non-researchers. Explain who (other than members of the research team) will have access to data (e.g., sponsors, advisers, government agencies, etc.).

The registers and forms are the property of the Zimbabwe MOHCC or ZiCHIRe and are not shared with researchers outside of those using routine Monitoring &Evaluation data for program evaluation and policy development, including the registers and forms used for this study. The medical records used at the facilities are owned by the service providers, including ZiCHIRe, and will not be shared.

4. Will you place a copy of the consent form or other study information in the subject’s medical or other personal record?

No  Yes. If yes, explain why this is necessary.

Zimbabwe MOHCC requires that the VMMC consent form be kept with the patient file.

5. Do you anticipate using any data (information, specimens, etc.) from this study for other studies in the future?  No  Yes

If “Yes,” explain and include this information in the consent form.

**H. ADDITIONAL INFORMATION**

1. If the study will involve radiation exposure to subjects, e.g., X-rays, radioisotopes, what is status of review by the UW Radiation Safety Committee (RSC):  Pending  Approved (Attach one copy of approval.)  NA

2. Does this research require approval from the UW Institutional Biosafety Committee (IBC) for recombinant/synthetic DNA human Gene transfer or vaccines?

No  Yes. If yes, what is the status of review by IBC?  Pending  Approved (Attach one copy of approval.)  NA

3. Protected Health Information (PHI). Will you or any member of your research team obtain, access, or use a subject’s protected health information by any method, and for any purpose including “pre-screening”?

*“Methods” may include but are not limited to: directly looking at a medical record (electronic or paper), requesting medical record information from a service such as the UW Center for Health Excellence, or viewing surgery schedules, clinic records, appointment books, etc.*

*Examples of where PHI may be located include: medical records, dental records, clinical lab tests that you will have performed on subject samples, pharmacy records, medical billing records, clinical databases, etc.*

No  Yes. If “yes”:

a. Describe the type of records/data, location and how you will obtain the information:

As OI clinics only see HIV+ patients, these activities do not require a review of confidential patient records. Recruiters at OI clinics and at study procedure clinics will ask patients for HIV test results as noted in their health passports or health records, including CD4 counts as recorded in these records. These records are commonly held by patients. Recruiters at Zengeza and Chitinguiza will also ask participants for this same information. These records are held by individuals and would be shared as part of the screening, eligibility, and consent process.

b. Will you obtain any of the information without HIPAA authorization from each subject?

No  Yes. If “yes”: Complete and attach the [Waiver Request: HIPAA Authorization](http://www.washington.edu/research/hsd/docs/853), and the [Waiver Request: Consent or Consent Requirements](http://www.washington.edu/research/hsd/docs/862). If the records are owned by the University of Washington or a state agency, complete and attach a UW Confidentiality Agreement.

HIPAA requirements do not apply in the setting we are conducting this study in, based on Zimbabwean common law. For this reason, a HIPAA waiver is not being requested. Clients who do not wish to share their health information may choose not to participate in the study.

c. Will you obtain HIPAA authorization from subjects for any of the information?

No  Yes. If “yes”, attach the HIPAA Authorization form you propose to use

d. Will you be obtaining any of the data as a Limited Data Set?

No  Yes

4. Other Records. Will you or any member of your research team obtain, access, or use academic, employment, or any other type of records about subjects, by any method, and for any purpose including “pre-screening”?

*“Methods” may include but are not limited to: directly looking at a record (electronic or paper), requesting records from*

*offices such as Payroll or the UW Registrar’s Office, obtaining records from the state Department of Health, etc.*

No  Yes. If “yes”:

a. Describe the type of records/data, location, and how you will obtain the information.

b. Will you obtain any of the information without the subject’s consent?

No  Yes.

If the records are owned by the University of Washington, complete and attach a UW Confidentiality Agreement.

5. Will you use the Clinical Research Center (CRC) at the UW or Seattle Children’s for any of your research activities?

No  Yes.

If you answered “yes”:

A medical record will be created for your subjects at UW Medicine and CRC staff may need to access those medical records for you. This may be because they are performing procedures or collecting data for you. It may also be required if an event happens on the CRC that requires treatment (such as fainting during a blood draw). This means that you must obtain a signed HIPAA Authorization form from each subject and give a copy of it to the CRC. ***Complete and attach the UW research HIPAA Authorization template, available on the HSD Forms webpage. There is guidance in the template about how to describe the information that the CRC staff may access and disclose to you.***

6. Will you make audio-visual or tape recordings or photographs of subjects?  No  Yes. If yes, explain what type of recordings you will make, how long you will keep them, and if anyone other than the members of the research team will be able to see them.

If the study participant consents, the circumcision procedure will be videotaped and photographed. Individuals can be part of the study whether or not they agree to be videotaped and photographed as noted in the consent. The penis will also be pictured in each follow-up visit to show the healing process. Photographs will be taken on the penis only, never the face, showing the study number, date of photographs and the study day. The photographs will make up part of the study record and not the subject’s permanent medical record. At the end of day, each photograph is downloaded on a protected computer. All photographs are kept in locked filing cabinetss and only members of the study team have access to them. These will be kept up to 5 years after the end of the study and then destroyed. The recordings will only be used for teaching, research and presentation at professional meetings..

7. Will your study involve use of equipment involving energy input to the subjects (EMG, EKG, MRI, ultrasound, etc.)?

No  Yes. If yes, attach documentation that all equipment will be tested regularly by the Scientific Instrument Division (call (206) 543-5580 for information) or describe safety testing procedures you will use.

8. Confirm by checking the box that the principal investigator on this IRB application has ensured that all investigators (as defined by [UW policy GIM 10](http://www.washington.edu/research/osp/gim/gim10.html)) are aware of policy GIM 10 and their responsibility for complying with its relevant requirements.

Confirmed

9. Does the individual who is the principal investigator on (1) this IRB application or (2) any grants or contracts supporting this research have a financial conflict of interest with respect to this research?  No  Yes.

10. If yes, has it been disclosed to the University? (Since August 24, 2012, all disclosures are made through the University’s online [Financial Interest Disclosure System](http://www.washington.edu/research/tools/systems/fids).) Final review of this application cannot occur until the disclosure has been made and reviewed by the University, and the outcome has been incorporated into the IRB’s review.  No  Yes  Not applicable, because there is no financial conflict of interest.

**I. CONSENT**

1. Description of consent process for adult subjects. How are you going to obtain informed consent from your adult subjects? Describe in detail your consent methods, process, and settings. Identify who will provide the information to subjects and who will interact with them during the consent process. If there is more than one consent process, describe each one separately. For subjects who do not speak English: Describe the process that will be used, and whether anyone on the research team will speak the subjects’ language. **If you are requesting a waiver of consent for all subjects and procedures, skip to the next section.**

Informed consent is the final step in the pre-VMMC counseling process. All HIV+ men over age 18 who present to participate in the study will undergo the following counseling and consent process as indicated in the attached documentation. The informed consent for VMMC clients is included in Appendix 1.

All clinicians who are conducting male circumcisions as part of the study will be eligible to participate in interviews at one point in time, approximately 4 weeks after implementation begins. . Clinician participation is voluntary; those who refuse participation will continue conducting circumcisions as part of the study. The informed consent form for clinicians is included in Appendix 2.

2. Description of assent process for children subjects. Describe in detail how you will obtain assent from children subjects, following the instructions provided in the question above. Also, describe how these processes will differ based on age/cognitive ability. Finally, describe how you will determine whether a child is assenting or dissenting throughout the research (if applicable).

No men under age 18 will be enrolled in this study.

3. Special issues or considerations. The standard concept of consent is based on the Western ethical tradition of individual autonomy and privacy. This may not apply well to your research. Your research may be subject to specific cultural or other contextual issues that affect the consent process. Describe any special issues and considerations about obtaining consent for your research. If none, state: “Not Applicable”.

Example issues:

- Who is the appropriate person(s) for providing consent?
- The desirability of a group consent process, or a surrogate consent process
- Research that occurs in a setting with a blurred sense of what is public versus private
- The cultural acceptability of the consent process (or documentation)
- Cultures or groups in which it is considered impolite to refuse a request and/or in which people are fearful of refusing requests that they regard as coming from authorities

Not applicable

4. Undue influence. Describe how you will minimize any undue influence on your subjects’ decision about participating in your research. If this is not an issue for your research, describe why. *This is an important consideration when persons recruiting or consenting subjects are in a position of authority or influence – for example, the subject’s teacher, doctor, or employer.*

We will prevent undue influence by stressing the voluntary nature of this study and each man’s ability to decline the procedure during the recruiting, counseling, consent, or preparatory phases. We will also inform participants that they will not lose their access to health care or VMMC services if they decline to participate in this study.

5. Subject comprehension. Describe anything that you will do to facilitate or verify your subjects’ comprehension of the information you provide them during the consent process.

Information will be available for clients in English and in local languages.

6. Do you expect that all of your participants will be **fluent** in spoken and/or written English?  No  Yes.

If “No”, please answer the following questions.

6.1. In what language(s) will they be fluent?

Shona.

6.2. Translation of documents into another language. Federal regulations require that consent, assent, and authorization documents must be presented to participants in a language that is understandable to them. The UW IRB expects that translated documents will be:

- Linguistically accurate;
- At an appropriate reading level for the subject population; and
- Culturally sensitive for the locale in which they will be used.

Describe how you will obtain translations of relevant documents, and how you will ensure that the translations meet these requirements.

All documentation will be drafted in English and translated by bilingual study staff. Consent forms, advertisements, and study information materials will be translated by the local study team. Interviewer-administered forms will be used in English, as used in previous studies under the Zimbabwean protocol. Trained interviewers translate orally from English to Shona and complete forms in English. All study forms will be completed in English by study staff.

6.3. Interpretation. Describe how you will provide interpretation, and when. Specifically:

1. For what situations will you provide interpretation? (At a minimum, an interpreter should be available for the consent process, unless the IRB has waived consent.)

The study will not use any interpreters.

1. Who will be the interpreter?

1. Describe the qualifications of the interpreter – for example, background, experience, language proficiency in English and in the other language, native language fluency, certification, other credentials, familiarity with the research-related vocabulary in English and the target language.

1. How will you ensure that the subjects will understand ongoing study-related communication? If the subject has questions, complaints, or adverse events, how will that be communicated to the researchers?

*Check all that apply:*

**Written** *Attach copies of all consent forms for each subject group. Include a footer identifying the version date of each form and a header or title that identifies each different form. If you propose to delete one or more of the required elements of consent from a consent form, attach and complete the form called* [*Waiver Request: Consent or Consent Requirements*](http://www.washington.edu/research/link.php?id=1595)*.*

**Waiver of written documentation of consent** *This means that you are requesting a waiver of the requirement to obtain written documentation of consent. Complete and attach the form called* [*Waiver Request: Consent or Consent Requirements*](http://www.washington.edu/research/link.php?id=1595)*. Also, attach the Information Statement, oral consent or assent protocol and script, or other materials you will use to communicate the necessary elements of consent to the subjects.*

**Waiver of consent** *This means that you are requesting a waiver of the requirement to obtain consent. Complete and attach the form called* [*Waiver Request: Consent or Consent Requirements*](http://www.washington.edu/research/link.php?id=1595)*.*

**Assent** *Attach copies of any written materials or scripts you will use with minor subjects (individuals under the age of 18) to obtain their assent to being in your research.*

**Parental permission** *Attach copies of any written materials or scripts you will use with parents, to obtain their permission to enroll their minor children in your research. See also* [*Supplement: Protected and/or Vulnerable Populations*](http://www.washington.edu/research/hsd/docs/322) *for waivers or alterations of consent requirements.*

**J. DRUGS, SUBSTANCES, AND DEVICES**

1. List **all** non-investigational drugs or other substances used to conduct this research (analgesics, anesthetics, drugs used to treat side effects, etc.). Include products used for standard clinical care if they are used in this study for research purposes.

| Name | Source | Dose | How administered |
| --- | --- | --- | --- |
| Ibuprofen | ZiCHIRe will source locally | 400 mg | Oral |
| Lidocaine | ZiCHIRe will source locally | 5% dermal cream | Topical |
| Cloxacillin | ZiCHIRe will source locally | 250 mg | Oral |
| Ciprofloxacin | ZiCHIRe will source locally | 500 mg | Oral |
| Paracetamol | ZiCHIRe will source locally | 500 mg | Oral |

2. List **all** investigational new drugs or other investigational substances to be used in the study. Include marketed products used “off-label” (different formulation, dose, route of administration, or indication). Provide:

1. **three** copies of a concise summary of information about the drug prepared by the investigator (including animal and human toxicity data, studies done in animals and humans to date);
2. **one** copy of the Investigator’s Brochure;
3. **one** copy of the study protocol.

**Important note**: You must register an IND with the appropriate institutional pharmacy (**UWMC: 598-6054; HMC: 731-5448,
VA: 764-2142**) before using the drug in research.

| Name | Source | Dose | How administered | IND Number | Phase of testing |
| --- | --- | --- | --- | --- | --- |
|  |  |  |  |  |  |
|  |  |  |  |  |  |
|  |  |  |  |  |  |
|  |  |  |  |  |  |

3. List all investigational devices you will use. Provide the information requested below and attach one copy of the company protocol. If there is no Investigational Device Exemption (IDE), explain why. Include a statement as to why the device qualifies as non-significant risk. Provide a copy of the FDA letter(s) which states the device classification (PMA, 510K, Class I, II, or II, or custom device) and categorization (Category A or B). “Category A” means that Medicare may **not** be billed for the device or for services related to its use. “Category B” means that Medicare may be billed for services related to its use **if** the U.S. Health Care Finance Administration (HCFA) grants authorization. **Important Note:** Register IDE devices with the UWMC Manager of Surgical Support Services (598-6538) or the HMC Business Manager of Surgical Services (731-8094) to obtain authorization for use.

a. Name of the device: PrePex™

The PrePex™ device is FDA cleared with 510(k) status (K103695; see 510(k) and is thus not considered “investigational”; however we are providing the following details for the information of the IRB review committee. The device is certified CE Mark Class IIa. It is manufactured using USP Class VI biocompatible elastomeric materials compliant to ISO_13485 Medical Devices (Quality Management systems) and FDA, 21 CFR177. 2600. Circ MedTech is ISO 13485 certified.

b. Name of the manufacturer: Circ MedTech

c. Description of its purpose and how you will use it in this study:

PrePex™is a single use, disposable device; indicated for circumcision of adult men, defined as circumferential excision of the foreskin or prepuce at or near the level of coronal sulcus, with minimal amount of preputial skin remaining.

d. Descriptions of previous studies in humans and animals:

The WHO Technical Advisory Group (TAG) on Innovations in Male Circumcision reviewed eight studies from 3 African countries in the WHO Framework for Clinical Evaluation of Devices (see WHO Pre-Qualification Report: <http://www.who.int/diagnostics_laboratory/male_circumcision/130530_pqmc_public_report_final_v1.pdf>). All studies included only healthy males 18 years and older. The device was placed successfully on 2417 eligible men. Adverse events occurred among 1.7% of participants; the majority were Mild and Moderate, while 0.4% were considered Serious as prompt surgical intervention was required to prevent serious long-term sequelae (in all cases surgical intervention was successful with no complications and satisfactory outcomes). The AE rate was lower among men who had PrePex™ circumcision than among men who had conventional surgery, but this difference was not statistically significant. No mechanical failures were reported. Healing time appears to be about 1 – 2 weeks longer than following conventional surgical circumcision. Some pain occurs with PrePex™ procedures, primarily during the first few days after placement and briefly on removal of the device. Complaints of unpleasant odor were also noted by some study participants and providers.

The WHO Technical Advisory Group (TAG) on Innovations in Male Circumcision concluded that the range and scope of clinical studies met the WHO requirements for evaluation of a device; and demonstrated that, for the purposes of HIV prevention, the PrePex™ device can efficaciously and safely circumcise healthy males over 18 years, when used by suitably trained providers, and when surgical backup facilities and skills are available to manage device displacements or early removals that could result in serious complications.

The TAG advised that: effective training materials are available and PrePex™ providers are appropriately trained; men receive accurate information on safe device use and risks while wearing a device and PrePex™ is used only in settings where suitable surgical facilities and skills are available within a short time frame (6-12 hours). This conclusion is time-limited and must be reassessed in about one year when more experience and data are generated to further inform safe use.

e. Investigational Device Exemption (IDE) number or FDA status:

WHO Prequalification Number: PQMC 0001-001-00

FDA 510(K) K103695 – Substantially Equivalent: <http://www.accessdata.fda.gov/cdrh_docs/pdf10/K103695.pdf>

Appendices:

Appendix 1: Consent Form – Men

Appendix 2: Consent Form – Clinician

Appendix 3: Standard Operating Procedures – Clinical Team

Appendix 4: Case Reporting Form – Screening

Appendix 5: Case Reporting Form – PrePex Device

Appendix 6: HIV+ Male Questionnaire – Day 0

Appendix 7: HIV+ Male Questionnaire – 2 Weeks Post Procedure

Appendix 8: HIV+ Male Questionnaire – 90 Day Post Procedure

Appendix 9: Clinician Questionnaire

Appendix 10: Recruiting Script

Appendix 11: Project Proposal

Appendix 12: Notice of Award – Budget

Appendix 13: Copy of Zimbabwe IRB Approval, 2012

Appendix 14: Copy of Zimbabwe IRB Approval, 2013

Appendix 15: Prepex manufacturer instructions for hygiene

Appendix 16: Recruiting flyer
